# *Bt*-Modified Transgenic Rice May Shift the Composition and Diversity of Rhizosphere Microbiota

**DOI:** 10.3390/plants13101300

**Published:** 2024-05-08

**Authors:** Qixing Huang, Yuliang Zhang, Yanhua Tan, Hua Kong, Yang Cao, Jungang Wang, Guohua Yin, Anping Guo

**Affiliations:** 1 National Key Laboratory for Tropical Crop Breeding, Institute of Tropical Bioscience and Biotechnology, Chinese Academy of Tropical Agricultural Science, Sanya 572024, China; huangqixing@itbb.org.cn (Q.H.); zhangyuliang@itbb.org.cn (Y.Z.); caoyang@itbb.org.cn (Y.C.); wangjungang@itbb.org.cn (J.W.); 2 Hainan Key Laboratory for Biosafety Monitoring and Molecular Breeding in Off-Season Reproduction Regions, Sanya Research Institute of Chinese Academy of Tropical Agricultural Sciences, Sanya 572024, China; 3 National Plant Protection Observation and Experiment Station at Sanya, Institute of Tropical Bioscience and Biotechnology, Chinese Academy of Tropical Agricultural Science, Sanya 572024, China; 4 College of Biological and Chemical Engineering, Qilu Institute of Technology, Jinan 250200, China; 5 Department of Plant Biology, Rutgers, The State University of New Jersey, New Brunswick, NJ 08901, USA

**Keywords:** genetically modified rice, microbial communities, 16S ribosomal RNA, transgenic *Bt* rice variety, operational taxonomic units

## Abstract

Plants significantly shape root-associated microbiota, making rhizosphere microbes useful environmental indicator organisms for safety assessment. Here, we report the pyrosequencing of the bacterial 16S ribosomal RNA in rhizosphere soil samples collected from transgenic *cry1Ab/cry1Ac Bt* rice Huahui No. 1 (GM crop) and its parental counterpart, Minghui63. We identified a total of 2579 quantifiable bacterial operational taxonomic units (OTUs). Many treatment-enriched microbial OTUs were identified, including 14 NonGM-enriched OTUs and 10 GM-enriched OTUs. OTUs belonging to the phyla Proteobacteria, Actinobacteria, Acidobacteria, Firmicutes, Nitrospirae, Chlorobi and GN04 were identified as statistically different in abundance between GM and the other two treatments. Compared with the different impacts of different rice varieties on microbiota, the impact of rice planting on microbiota is more obvious. Furthermore, Huahui No. 1 transgenic *Bt* rice had a greater impact on the rhizosphere bacterial communities than Minghui63. Early developmental stages of the transgenic *Bt* rice had a significant impact on many Bacillaceae communities. Soil chemical properties were not significantly altered by the presence of transgenic *Bt* rice. The peak concentration level of *Bt* protein products was detected during the seedling stage of transgenic *Bt* rice, which may be an intriguing factor for bacterial diversity variations. Based on these findings, we conclude that transgenic *Bt* rice has a significant impact on root-associated bacteria. This information may be leveraged in future environmental safety assessments of transgenic *Bt* rice varieties.

## 1. Introduction

Rhizosphere microbiota often perform important ‘external’ functions for plants, including nutrient cycling, induction of the immune system, and pathogen antagonism [1,2]. Numerous studies have shown the ability of beneficial microorganisms to improve plant growth and health through the production of stimulatory compounds and direct interactions with hosts [2,3,4]. In turn, plants actively secrete plant compounds that specifically stimulate or repress microorganisms, leading to changes in their rhizosphere microbiome. Referred to as the ‘second genome of the plant’, this plant-associated microbial community is continuously being shaped by the plant [5]. Therefore, it is not surprising that the type of plant species and its developmental stage will have a significant impact on the composition of the rhizosphere microbiome [6,7].

Next-generation sequencing technologies in recent years have enabled further characterization of plant-associated microbial community structure, function, and ecological roles. Bacterial diversity studies have successfully utilized the 16S rRNA-based metagenomic analyses to study the plant rhizosphere microbiome [8]. The application of these methods includes performing barcoding strategies and targeting specific regions through the usage of large-scale Sanger sequencing, oligonucleotide microarrays, and 454 pyrosequencing. Metagenomic studies enable both the comprehensive comparisons of microbial community profiles and a better understanding of the potential impact of specific microbial communities. For example, plant root-associated microbiota from several *Arabidopsis* species and their impacts on environmental factors were studied using high-throughput metagenomics DNA sequencing [9,10,11]. These studies show that the composition of root microbiota interactions required balancing environment-specific host needs, host genome variations, and the presence of microbe populations within a specific soil type. 

There is a growing interest in understanding the ecological impact of genetically modified (GM) crops on soil ecosystems and microbiome compositions. As of 2019, GM crops have been adopted in 29 countries, covering an area of more than 190.4 million hectares [12]. The presence of insecticidal genes (*cry* genes) from *Bacillus thuringiensis* (*Bt*) in GM corn and GM potatoes has led to a significant increase in yield and reduction in pesticide usage [13,14]. In order to address the needs of the food supply, land degradation problems and chronic water shortages, the Chinese government initiated a $3.5 billion GM crop initiative project to research and develop GM plants [15]. Early studies in China were promising, and planting pest-resistant crops such as transgenic *Bt* rice has provided benefits to farmers by reducing pesticide use and labor input [16]. However, addressing the increasingly stringent guidelines set by the consumer and regulatory authorities to evaluate and assess the safety of genetically modified organisms in the environment remains a major challenge in GM crop development [17]. 

Earlier studies assessing transgenic crops in agricultural conditions demonstrated that transgenic plants did not cause significant changes in soil microbial communities compared to non-transgenic crops [17]. The transgenic rice lines Huahui No. 1 and *Bt* Shanyou63 (event TT51-1) were found to be resistant to insect pests, with excellent agronomic performances, and became the first approved transgenic rice in China in 2009 [17]. Environmental assessments of these transgenic rice lines were strictly performed, and it was concluded that they were safe and friendly to the surrounding biodiversity [18]. Similarly, when compared to wild-type varieties, several transgenic *Bt* crops did not significantly affect microbial compositions nor microbial activities in the rhizosphere during crop development [19,20,21,22]. However, in a recent study, reduced concentrations of phenolic compounds and root exudates in transgenic rice led to a decrease in rhizospheric bacterial diversity, illustrating the importance of microorganisms and their interactions with allelochemicals in soil [23]. Currently, there is limited information on the ecosystem biochemical cycling and bioactivity of transgenic plants and their metabolites. It is also becoming increasingly important to determine the environmental safety of GM crops due to contradictory reports of transgenic plants and their potential impact on non-target organisms and biological significance [17,24]. 

In this study, we present a quantitative evaluation of rhizosphere bacterial diversification between transgenic *Bt* rice line Huahui No. 1, and its parental non-transgenic rice line Minghui63, grown under field conditions and during different rice developmental stages. We identified many treatment-specific bacterial populations. To our knowledge, this is the first reported evidence of microbial variances induced by the presence of *cry1Ab/cry1Ac* Huahui No. 1 rice. We found that the concentration level of exogenous *Bt* protein products may be a key factor affecting bacterial variations. These findings provide new insights into the bacterial variation caused by planting GM rice, which may be leveraged in future environmental safety assessments of transgenic *Bt* rice varieties.

## 2. Results

### 2.1. Defining Abundant Community Members

We generated 466,923 raw reads from 72 samples (Supplementary Table S1). A total of 220,622 high-quality sequences were included in the subsequent analysis, ranging from 1638 to 7001 high-quality sequences (with a median of 2889). The taxonomic designation of each OTU representative sequence is shown in Supplementary Table S2. A total of 2579 unique OTUs were identified across all samples.

Similar to a previous study by Schlaeppi et al. [11], the threshold-independent community (TIC) was determined by a sampling depth of 1600 sequences per sample, including 2549 OTUs (Supplementary Table S3 and Dataset S1). Among the OTUs assigned in the TIC dataset, 1266 OTUs (49.67%) were low-abundance (<10 sequences). Non-reproducible OTUs were used for rarefaction analysis (Supplementary Figure S1), suggesting that low-count OTUs contribute to the microbiome richness. So, subsequent analyses were mainly focused on abundant community members (ACMs). The ACMs were represented by 168 bacterial OTUs, including 55.78% of rarefied sequences (Supplementary Table S4 and Dataset S2).

### 2.2. Community Composition Defined by Different Treatments

All ACM OTUs were taxonomically classified to the bacteria domain (Supplementary Table S4 and Figure S2). Many OTUs belonged to Proteobacteria (13.33%, 10.91%, 6.67% and 5.45% in the Deltaproteobacteria, Gammaproteobacteria, Alphaproteobacteria and Betaproteobacteria classes, respectively), Firmicutes (18.79%), Actinobacteria (15.76%) and Acidobacteria (14.55%). Similar taxonomic characteristics were also found in TIC samples (Supplementary Table S3 and Figure S2). We noted the dominance of *Bacillus flexus* (OTU1) in root communities of all samples (8.25% of ACM total community). A high OTU diversity within certain orders such as Actinomycetales (101 OTUs in 21 families) and Clostridiales (237 OTUs in 16 families) were also observed in TIC samples (Supplementary Table S3).

**Figure 1:**
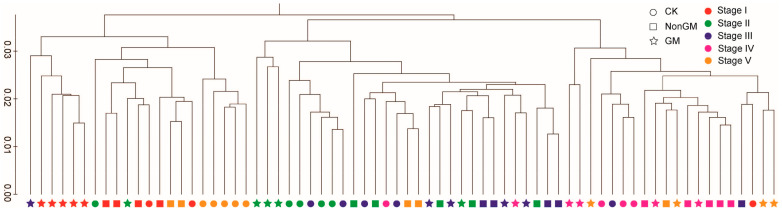
Beta diversity of the ACM. Between-sample diversity was calculated for ACMs using the weighted UniFrac distance metric (phylogeny-based and sensitive to the sequence abundances) on 800 sequences per sample.

The hierarchical clustering results show that both host species and sampling times contributed to variation in ACMs (Figure 1) and in TICs (Supplementary Figure S3). These variations measured by UniFrac distances were significantly different (*p* < 0.05). No consistent clustering was detected, either by treatment or by sampling time, indicating that the beta diversity was obscured by variation between samples. However, further network analysis between samples and ACM OTUs (relative abundance ≥ 20) show that both the GM and CK samples (for convenience, we labelled the control group as “CK”) contained enhanced numbers of treatment-specific OTUs (Supplementary Figure S4), indicating an enrichment bias of bacterial species by the GM and CK treatments.

Estimations of OTU diversity from OTU richness and Faith’s Phylogenetic Diversity metric [25] show that GM ACMs were of lower diversity and richness compared to the CK and NonGM microbiota (Supplementary Figure S5). However, these differences were not obvious and not detected in the TICs (Supplementary Figure S1). These findings further support the fact that qualitatively different treatment-associated bacteria existed among the GM, NonGM and CK treatments.

Results from analyzing ACM OTU variations in mean relative abundance across samples belonging to different treatments (Supplementary Table S6) show that between the CK and GM, CK and NonGM, and GM and NonGM treatments, 98 (58.33%), 54 (32.14%) and 19 (11.31%) OTUs were significantly different (FDR < 0.05), respectively. The abundances of many bacterial species in the root of GM rice were not consistent with those in NonGM and CK. Moreover, compared to CK, GM and NonGM were more consistent in OTU abundance, indicating the existence of a relatively stable microbiota in rice paddy fields.

**Figure 2:**
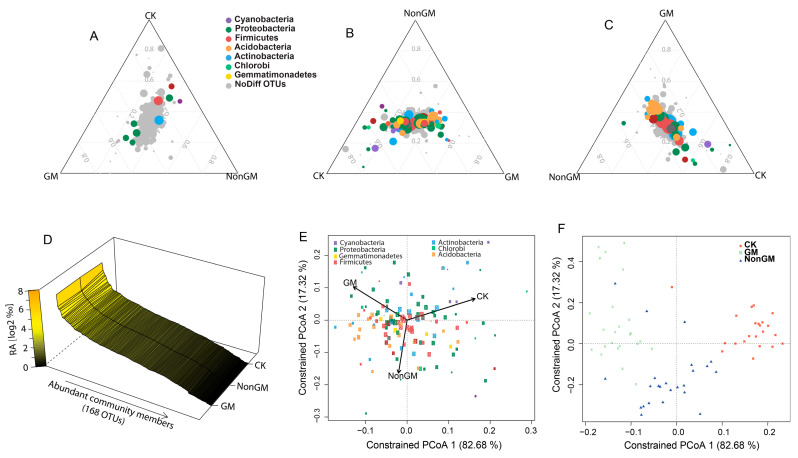
Root microbiota comparisons of GM, NonGM, and CK. The ternary plots depicted the relative occurrence of individual OTUs (circles) in samples of GM and NonGM compared with CK samples (**A**), in samples of GM and CK compared with NonGM samples (**B**), and in samples of NonGM and CK compared with GM samples (**C**), respectively. Colored circles depict significantly different (FDR < 0.1) OTUs between two treatments at the bottom of the triangles. The mean abundance of individual OTUs was calculated in each treatment and plotted ranked by average OTU abundance across all treatments (**D**). OTU scores of principle coordinate analysis of ACM OTUs (**E**) and scores of principle coordinate analysis for all samples based on Bray–Curtis distance (**F**), constrained by treatments and based on Bray–Curtis compositional dissimilarities among all samples. The arrows pointed to the centroid of the constrained factor. Circle size corresponded to relative abundance of OTUs/samples, and colors were assigned to different phyla. The percentage of variation explained by each axis referred to the fraction of the total variance in the data explained by treatments.

Then, quantitative different individual community members were identified between treatments. Compared to CK, a set of nine OTUs with marginally significant different abundances (Tukey, *p* < 0.1) was found in either the GM or NonGM treatments (Figure 2A), consisting of five Proteobacteria, one Firmicutes, one Actinobacteria, one GN04 and one Nitrospirae OTU. The abundances of five OTUs (including a Bacillales OTU64) were significantly higher in NonGM (FDR < 0.1), while another four OTUs were of significantly higher abundance in GM (FDR < 0.1) (Supplementary Figure S6). Additionally, 68 ACM OTUs with significantly different abundance (FDR < 0.1) between GM and CK (Figure 2B), and 40 ACM OTUs with significantly different abundances (FDR < 0.1) between NonGM and CK (Figure 2C) were also found. These findings revealed that many bacterial communities with similar abundances were shared in GM and NonGM rice paddy fields.

Community structures with qualitative similarities among all three treatments were shown by rank abundance profiling of the 168 ACM OTUs, indicating that microbiota variation was largely quantitative (Figure 2D). The Canonical Analysis of Principal (CAP) analysis [26] was used to investigate the contribution of significantly different ACM OTUs (FDR < 0.1) (Figure 2E), and samples of different treatments (Figure 2F), to the overall variation in all three treatments. Along the first principal coordinate, a clear differentiation was detected between treatments with the largest fraction of variation (82.68%). We confirmed the variation in soil microbiota was largely dependent on the host species. Importantly, we noted that the GM OTUs and samples contributed a large part to the coordination space formation, indicating that the planting of GM *Bt* rice contributed to a greater diversity of soil microbiota. 

### 2.3. Community Composition Defined by Different Sampling Times

Quantitative analysis of bacterial community compositions defined by sampling times within and between treatments to investigate rice development stages found very few bacterial OTUs to be significantly different in relative abundance between the developmental stages of GM and NonGM rice (Supplementary Figure S7). Furthermore, many more bacterial OTUs were significantly different (FDR < 0.1) in abundance between GM and CK (Supplementary Figure S8), and between NonGM and CK (Supplementary Figure S9), evidencing that the abundance of bacterial assemblies was predominantly changed by planting rice.

Interestingly, between stages I and II, we noticed that many more OTUs differed in abundance between CK and GM (Supplementary Figure S8) compared to the situation between CK and NonGM (Supplementary Figure S9). These OTUs were mainly classified into the phyla Proteobacteria, Firmicutes, Actinobacteria and Acidobacteria. Results of ACM OTU scores by principal coordinate analysis further confirmed the difference between stages I and II (Supplementary Figure S10). The largest fraction of the variation (66.72%) in both samples and OTUs in GM treatment could be detected between stages I and II. These findings provide evidence for the existence of a greater influence on root bacterial abundance caused by planting GM rice than by planting NonGM rice, especially in the fast-growing stages I and II.

Focusing on the analysis of mean relative abundance changes of all ACM OTUs between stages I and II, we found that 57 (33.93%) GM ACM OTUs (Supplementary Table S7) and 18 (10.71%) NonGM ACM OTUs (Supplementary Table S8) were significantly (FDR < 0.05) changed in abundance, while none of the CK ACM OTUs (Supplementary Table S9) were significantly changed in abundance. 

**Figure 3:**
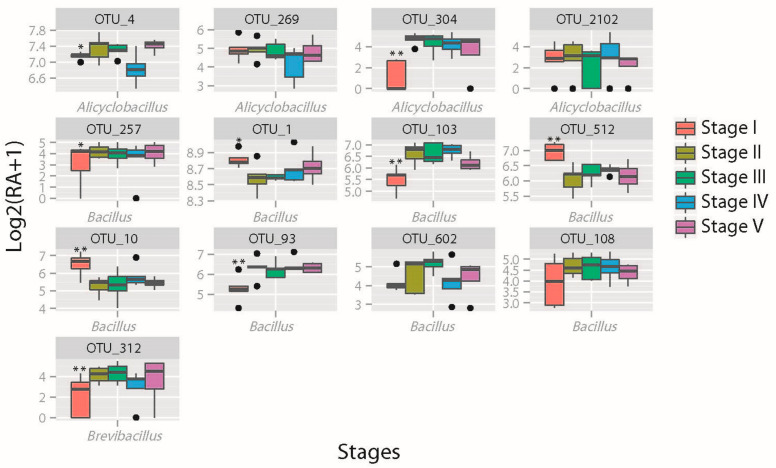
Stage-specific accumulations of thirteen Bacillaceae ACM OTUs in GM rice. Note: Nine Bacillaceae OTUs were changed significantly (FDR < 0.05) in GM ACM between stage I and stage II. OTU103\512\93\304\312\10 were very significant between Stage I and Stage II (FDR < 0.01). OTU4\1\257 were significant between Stage I and Stage II (FDR < 0.05). OTU4 was also significant between Stage III and IV, and between Stage IV and V (FDR < 0.05). RA, relative abundance; *, significant difference (FDR < 0.05); **, very significant difference (FDR < 0.01).

When we further investigated the changes in mean relative abundance in all 13 Bacillaceae ACM OTUs, results show that the abundance of nine Bacillaceae OTUs were changed significantly (FDR < 0.05) in GM ACM between stage I and stage II (Figure 3), including six OTUs (OTU10, OTU103, OTU304, OTU312, OTU512 and OTU93) of very significant difference (FDR < 0.01), and three OTUs (OTU1, OTU4 and OTU257) of significant difference (FDR < 0.05) in abundance change (Supplementary Table S7). Among these OTUs, abundances of three OTUs (OTU512, OTU10 and OTU1) increased while abundances of all the other six OTUs decreased from stage I to stage II. However, no significant changes in abundance relating to NonGM ACM and CK ACM Bacillaceae OTUs were observed (Supplementary Tables S8 and S9).

### 2.4. Identification of the Core Microbiota

We identified 40 ACM OTUs shared in all three treatments (Supplementary Figure S11A). These shared OTUs belonged to the phyla Proteobacteria (17 OTUs), Actinobacteria (8 OTUs), Acidobacteria (3 OTUs), Firmicutes (3 OTUs), Gemmatimonadetes (1 OTU), Cyanobacteria (1 OTU), Chloroflexi (1 OTU), and Chlorobi (1 OTU), and there were two unassigned OTUs (Supplementary Figures S11B and S12). These OTUs were statistically identical in abundance (FDR > 0.1) among treatments (Supplementary Table S6). Clustering analysis using these forty OTUs showed that samples from different treatments were basically of the same tendency in abundance change (Supplementary Figure S13). The contribution of the 40 core ACM OTUs to the overall variation in all three treatments was investigated by CAP analysis. We observed a differentiation between treatments, which explained a much smaller fraction of variation (52.67%) (Supplementary Figure S14) when compared with the aforementioned results (Figure 2E,F).

### 2.5. Identification of the Rice-Enriched Microbiota

We identified 54 ACM OTUs of significantly different abundance (FDR < 0.1) both in the GM and NonGM treatments as compared with CK (Supplementary Figure S11A). These OTUs belonged to the phyla Proteobacteria (13 OTUs), Actinobacteria (10 OTUs), Acidobacteria (10 OTUs), Firmicutes (9 OTUs), Cyanobacteria (3 OTU), Nitrospirae (3 OTUs), Chlorobi (2 OTUs), Chloroflexi (2 OTUs) and Gemmatimonadetes (1 OTU), and there was one unassigned OTU (Supplementary Figure S11C). These OTUs were statistically identical in abundance (FDR > 0.1) between GM and NonGM treatments (Supplementary Table S6). Clustering analysis of all samples using these OTUs clearly separated most of GM and NonGM samples from CK samples (Supplementary Figure S15). Amongst these OTUs, 14 rice-enriched OTUs with significantly higher abundances (change ratio ≥1.5-fold and FDR<0.05) were identified (Supplementary Figure S16). These 14 OTUs belong to five bacterial phyla, including Acidobacteria (OTU30, OTU47, OTU73, OTU79, OTU106 and OTU157), Actinobacteria (OTU45 and OTU815), Firmicutes (OTU98), Nitrospirae (OTU3, OTU28 and OTU102) and Proteobacteria (OTU14 and OTU29).

Sixteen CK-enriched OTUs with significantly higher abundances (change ratio ≥ 1.5-fold and FDR < 0.05) were identified (Supplementary Figure S17). These OTUs belonged to five bacterial phyla, including Acidobacteria (OTU23, OTU46, OTU61, OTU67 and OTU281), Chlorobi (OTU164 and OTU423), Cyanobacteria (OTU163, OTU177 and OTU368), Firmicutes (OTU269 and OTU512) and Proteobacteria (OTU74, OTU170 and OTU29), and one OTU (OTU116) belonged to an unknown phylum. Compared to CK-enriched OTUs, the identified rice-enriched core microbiota comprised Actinobacteria and Nitrospirae.

### 2.6. Defining and Characterizing GM Rice-Enriched Microbiota

**Figure 4:**
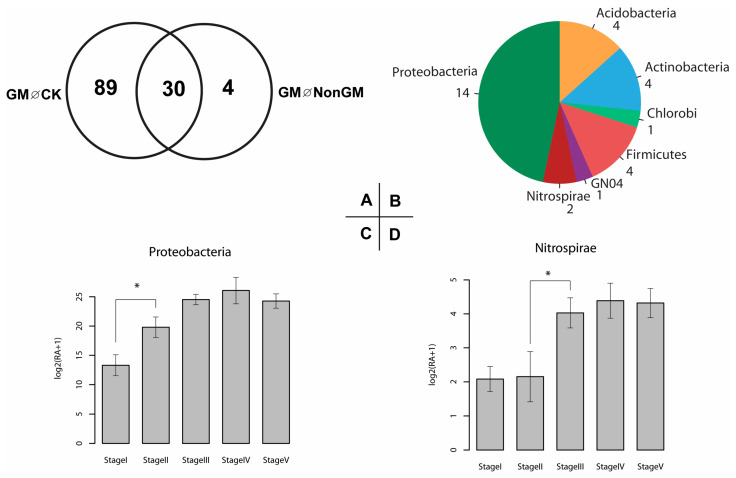
Identification and classification of significantly differed GM bacterial communities. Abundance of thirty OTUs was significantly different in GM, comparing with both CK and NonGM (**A**). Taxonomical profiles at the phylum rank and number of OTUs were shown (**B**). Proteobacteria OTUs were found to be significantly more abundant in stage II than in stage I (**C**), and Nitrospirae OTUs were found to be significantly more abundant in stage III than in stage II (**D**). ‘GM ∅ CK’ and ‘GM ∅ NonGM’ stand for significant OTUs (FDR < 0.1) between GM and corresponding treatments. *, significant difference (FDR < 0.05).

We found thirty ACM OTUs to be statistically different in abundance (FDR < 0.1) between GM and the other two treatments (Figure 4A and Supplementary Table S6). These OTUs belonged to the phyla Proteobacteria (14 OTUs), Actinobacteria (4 OTUs), Acidobacteria (4 OTUs), Firmicutes (4 OTUs), Nitrospirae (2 OTUs), Chlorobi (1 OTU) and GN04 (1 OTU) (Figure 4B). Next, bacterial community compositions of these OTUs defined by sampling times in GM treatment were analyzed quantitatively. Firstly, the variations in mean relative abundance in phylum level were measured. Interestingly, OTUs assigned to phylum Proteobacteria were significantly different in abundance between stages I and II (FDR < 0.05) (Figure 4C). Meanwhile, OTUs assigned to the phylum Nitrospirae were significantly different in abundance between stages II and III (FDR < 0.05) (Figure 4D). Secondly, the variations in mean relative abundance of individual OTUs across sampling times were measured. We found 17 OTUs to be significantly different between stages I and II (FDR < 0.1) (Supplementary Figure S18). Of these, only five OTUs had higher abundances in stage I while twelve OTUs had higher abundance in stage II. Predominantly, abundances of two Bacillales OTUs (OTU11 and OTU18) were higher in stage II than in stage I (Supplementary Figure S18).

**Figure 5:**
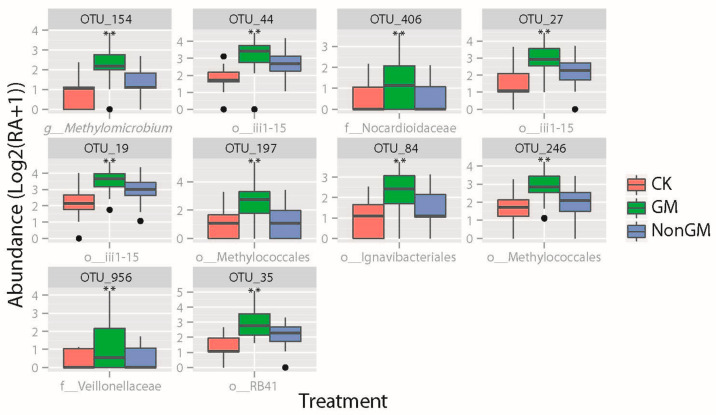
Abundance of ten GM-enriched OTUs. GM-enriched OTUs were defined as OTUs that are very significant in abundance (FDR < 0.01) between GM and CK, and between GM and NonGM. Minimum mean abundance log2(RA+1)) of GM OTUs ≥ 1. Comparing to CK and NonGM, the minimum increased changing fold of GM OTUs ≥ 1.5-fold. RA, relative abundance; f_, family; o_, order; g_, genus. **, very significant difference (FDR < 0.01).

A total of ten GM-enriched OTUs were further quantitatively defined (Figure 5). These GM OTUs displayed significant differences in abundance (FDR < 0.01) and at least a 1.5-fold higher abundance when compared with NonGM and CK treatments. These OTUs were assigned to the orders Actinomycetales (OTU406), Clostridiales (OTU956), Ignavibacteriales (OTU84), iii1-15 (OTU19, OTU27 and OTU44), Methylococcales (OTU154, OTU197 and OTU246) and RB41 (OTU35). Among these 10 GM-enriched OTUs, 6 OTUs were significantly different in abundance (FDR < 0.05) between stages I and II (Supplementary Figure S19). Clustering analysis of all samples using 10 GM-enriched OTUs clearly separated a greater part of GM samples from the other two treatments (Supplementary Figure S20).

### 2.7. ELISA Test of Cry1Ac Protein Level

**Figure 6:**
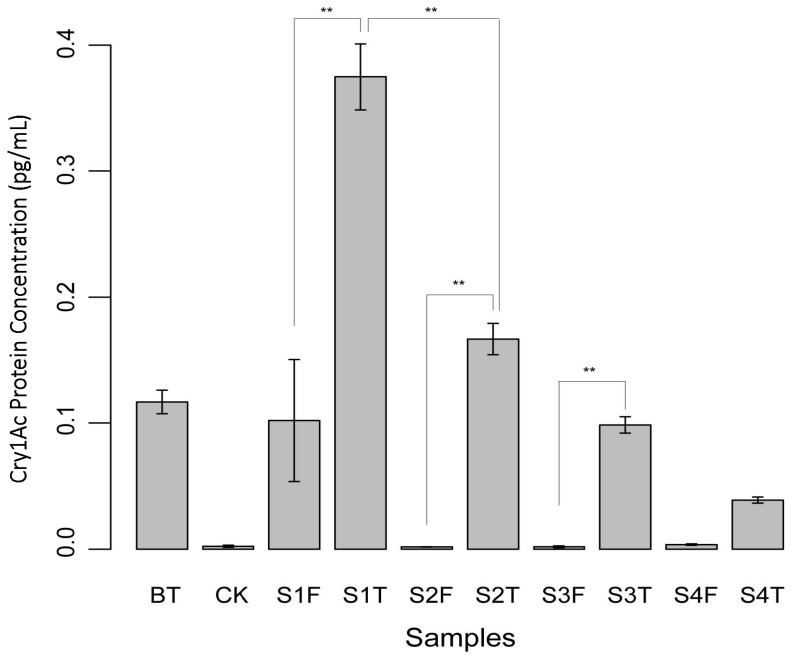
Concentration of Cry1Ac protein in Huahui No.1 transgenic Bt rice and its parental counterpart Minghui63 rice. The level of Cry1Ac protein was gradually decreased as rice was developing to higher stages. Bt, positive control; CK, negative control; S1, stage I; S2, stage II; S3, stage III, S4, stage IV; F, Minghui63 rice; T, Huahui No.1 transgenic Bt rice; **, very significant difference (FDR < 0.01).

The levels of cry1Ac Bt protein in GM rice across all developmental stages were measured by ELISA experiments (Figure 6). Results showed that the level of Cry1Ac protein in Huahui No. 1 transgenic Bt rice was significantly different (FDR < 0.01) to that in the non-transgenic Minghui63 rice. A decreasing tendency of the Cry1Ac protein level was detected with increasing stages. While 0.37 ± 0.03 pg/mL cry1Ac was found in Stage I GM rice seedlings, 0.17 ± 0.01 pg/mL cry1Ac was found in stage II tillering GM rice. No significant difference was detected in other stages.

### 2.8. DDGE Analysis of Bacterial Communities

**Figure 7:**
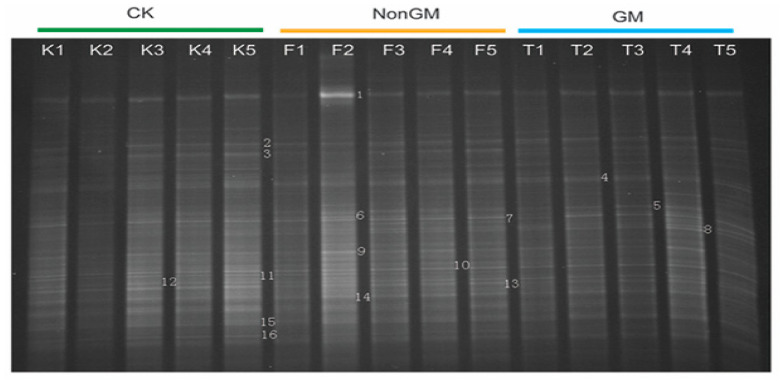
DDGE validation of 16S rDNA. Each column represents a pooled sample from the same treatment in same sampling time. Numbers adhered to gel bands corresponded to cloned sequences annotated in Supplementary Table S10. Note: K1~K5, CK samples from stage I to stage V; F1~F5, NonGM samples from stage I to stage V; T1~T5, GM samples from stage I to stage V.

We manually identified 16 differential electrophoretic bands (Figure 7). Based on the Ribosomal Database Project, these could be classified into four bacterial phyla including Proteobacteria (12 bands), Actinobacteria (2 bands), Firmicutes (1 band) and Acidobacteria (1 band) (Supplementary Table S10), which was in accordance with the predominant ratios of these four bacterial phyla in ACM and TIC samples (Supplementary Figure S2).

### 2.9. Comparison of Soil Properties between GM and NonGM Treatments

Results referring to the changes in soil chemical properties show that total phosphorus, total potassium and available phosphorus predominantly decreased between stages I and V in all three treatments (Supplementary Table S11). Using Duncan’s multiple range test method, no significant changes in the total nitrogen concentrations and available nitrogen were detected between different treatments at the 5% level. Results show that the planting of GM rice did not have a significant influence on soil properties in the short term, and it seems unlikely that the change in soil bacterial diversity was induced by the change in soil chemical properties.

## 3. Discussion

### 3.1. Rice Planting Tied More Strongly to Rhizospheric Microbiota Communities than Rice Variety

Recent studies have shown that the plant host and its developmental stage has a significant influence on the rhizospheric microbiome composition [6,7]. In this study, we examined soil samples collected from fields without rice (CK), wild-type Minghui63 (non-genetically modified (NonGM)), and transgenic Bt rice Huahui No.1 (GM). We found that the diversity of root-associated microbes is largely dependent on the presence of rice in the field. We also found significant differences in OTUs in GM and NonGM samples compared to CK (Figure 2). This is consistent with previous studies that reported the effects of root deposition of exudates, mucilage, and sloughed cells and their influence on the rhizosphere composition [1,27].

### 3.2. Transgenic Bt Rice Has a Stronger Impact on Rhizosphere Microbiota Communities, Particularly in the Early Developmental Stages of Rice

The abundance of many bacterial species in the root of GM rice significantly differed from those found in NonGM and CK conditions. The abundance was impacted by early developmental stages I and II, characterized by rapid plant growth (Supplementary Figures S7–S9). Planting Huahui No. 1 transgenic Bt rice had a stronger impact on the rhizosphere microbiota communities compared to wild-type Minghui63. Siciliano et al. demonstrated that root-associated microbial community compositions were different between transgenic and non-transgenic canola [28]. In contrast, Lottmann et al. did not detect differences in rhizosphere bacterial communities between transgenic T4-lysozyme-producing potatoes and its parental counterpart [29]. This contradictory evidence may be due to several factors, including differences in hosts and the introduction of different genes into the plants leading to differential effects in the composition of microbial communities. We found that planting Huahui No. 1 resulted in significant changes in microbial community abundance, including about 10% OTUs between the GM and NonGM treatments (Supplementary Table S6). Notably, the most predominant variations were detected between stage I and stage II of GM rice (Supplementary Table S7). These findings suggest that the active vegetative growth of GM rice in the early developmental stages (e.g., seedling and tillering stages) have the greatest impact on microbe diversity variations.

### 3.3. Huahui No. 1 Transgenic Bt rice Shows a Predominant Short-Term Impact on Bacillaceae Communities in Rice Early Developmental Stages

Bacillaceae communities are extensively distributed in soil environments. *Bacillus thuringiensis* in particular produces *Bt* proteins under natural growing conditions. Therefore, we hypothesized that planting Huahui No. 1 rice may increase the environmental content of *Bt* proteins, resulting in variations in Bacillaceae communities in soil. This study showed that planting Huahui No. 1 rice had a predominant short-term impact on Bacillaceae communities. In particular, we detected a significant increase in the concentration level of Cry1Ac protein (FDR < 0.01) in the early developmental stage (Figure 6).

Due to limited experimental conditions, we were not able to directly detect the Cry1Ac/Cry1Ab protein in rice roots and rhizosphere soil; we speculate that the change mode of *Bt* proteins in rice roots is likely to be consistent with that in rice leaves. The peak concentration level of *Bt* protein products in GM rice stage I seedlings (Figure 6) may likely lead to more *Bt* protein exudates from rice roots in the rhizosphere soil and have a stronger influence on Bacillaceae community diversity. Moreover, Bacillales microbes were significantly different between various developmental stages of GM rice. We propose that *Bt* proteins expressed by GM rice may be an important factor that impacts the variation in root bacterial diversity, especially during seedling and tillering stages. The selective pressure on *Bt*-sensitive soil bacteria would cause significant changes in population dynamics. Furthermore, the vigorous growing and cropping of GM rice may lead to the enrichment of Cry1Ab/Cry1Ac proteins in rice organic tissues; the release of these root exudates to the rhizosphere soil would lead to subtle changes in environmental conditions, ultimately affecting the bacterial diversity. 

*Bacillus flexus* (OTU1) was detected in all samples and contributed to 8.25% of ACM bacterial communities. Nine Bacillaceae OTUs were significantly different in abundance (FDR < 0.05) in the GM rice rhizosphere between stages I and II (Figure 3). In contrast, no significant change in abundance in the representative OTU of NonGM and CK (Supplementary Tables S8 and S9) was detected. It suggests that the Bacillaceae assemblies in conjunction with *Bacillus flexus* could be used as a biological indicator in future environmental safety assessments of GM *Bt* rice varieties. 

**Figure 8:**
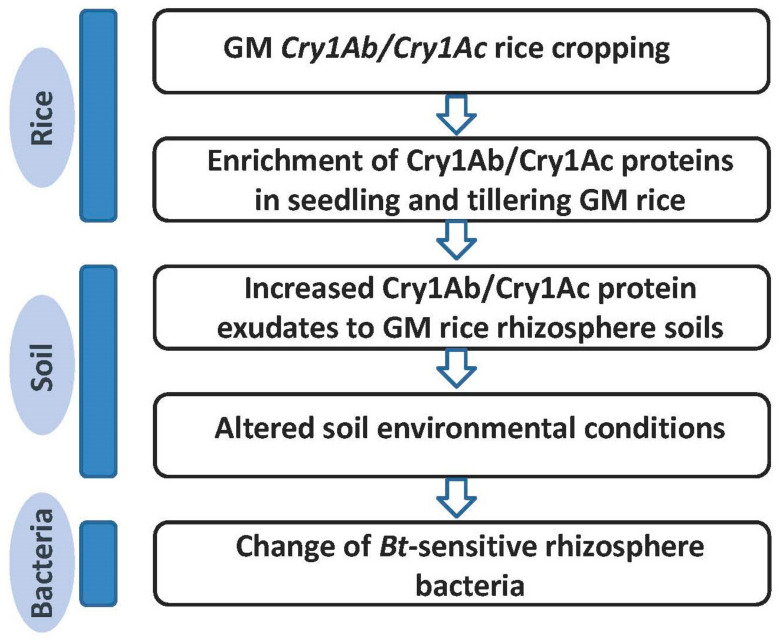
Hypothesized impact mechanism of planting GM *Cry1Ab/Cry1Ac* rice on rhizosphere bacteria.

Based on these findings, we infer a potential mechanism that might be involved in the establishment procedure: (i) GM *Bt* rice root-associate Bacillaceae bacteria respond to *Bt* protein level changes autonomously; (ii) a selective advantage for treatment-enriched members was gained by the interactions among microbes. Taken together, *CrylAb/Cry1Ac* GM rice had a significant short-term effect on Bacillaceae microbes, indicating that a long-lasting evaluation of GM rice on environmental microbiota is very necessary. Based on these studies, we postulated a model, as shown in Figure 8.

## 4. Materials and Methods

Root-associated soil samples were collected from paddy fields with Huahui No.1 transgenic *Bt* rice (event TT51-1, denoted as ‘GM’), paddy fields with Minghui63 rice (denoted as ‘NonGM’) and paddy fields with no rice planted (denoted as ‘CK’). Sampling times were designated according to different rice developmental stages after transplanting to paddy fields: seedling (18 days, denoted as ‘stage I’), tillering (61 days, denoted as ‘stage II’), panicle development (91 days, denoted as ‘stage III’), ripening (119 days, denoted as ‘stage IV’) and a post-harvest sampling time (145 days, denoted as ‘stage V’). Barcode sequence tags were specifically designed for each sample. The V6-V8 regions of the 16S rDNA gene was amplified from soil DNA with the primer pair V6F3: 5′CGTATCGCCTCCCTCGCGCCATCAG(barcode)TGCAACGCGAAGAACCTTACC3′ and V8R2:5′CTATGCGCCTTGCCAGCCCGCTCAGGCCCGGGAACGTATTCACCG3′, designed by Sangon Biotech (Shanghai, China). 

Pyrosequencing reads were processed and analyzed using USEARCH (version 8.0.1477) [30] and QIIME (version 1.9.1) [31] as described by Schlaeppi et al. [11], with some minor adjustments. Canonical Analysis of Principal coordinates (CAP) was performed in R (version 3.1.2) [26]. The threshold-independent community (TIC) and the abundant community members (ACMs) were defined and analyzed following analysis methods as reported by Schlaeppi et al. [11]. Rice leaves at four different rice developmental stages (stages I to IV) were collected and the *Bt* toxin protein productions were measured using the enzyme-linked immunosorbent assay (ELISA) method. Additionally, a denaturing gradient gel electrophoresis (DGGE) analysis was applied to detect variable bacterial communities. Furthermore, soil samples collected from stage I and stage V paddy fields were used to investigate the change in soil chemical properties. For more detailed methods, please see Materials and Methods in the included Supplementary Information.

## 5. Conclusions

In this study, we identified and characterized the treatment-specific microbiota for both rice-planting and GM treatments. Most importantly, we found that the cropping of Huahui No. 1 cry1Ab/cry1Ac transgenic *Bt* rice showed greater soil microbial variations. The transgenic *Bt* rice significantly impacted Bacillaceae communities, particularly in early developmental stages. This study recommended a model to summarize and suggest *B. flexus* to be used as an indicator species for a long-lasting evaluation of GM rice on environmental microbiota. Studying transgenic rice and their impact on rhizosphere microbiota can be influenced by limitations of the experimental conditions, such as the 16S rDNA PCR primer bias, the soil type, and the number of environments tested. Furthermore, using pyrosequencing and OTUs poses some limitations compared to using Illumina sequencing and ASVs for analyzing the microbiome. However, our data suggest common selective pressure dominated the microbial communities within the same habitat. Future root–microbiota studies should integrate metagenomics, metatranscriptomics and metaproteomics analyses to not only examine microbiota taxonomic lineages, but also their functions at the molecular level. These studies should encourage people to know more about microbe–microbe and microbe–host interactions [32,33]. We hope our findings will not only aid in exploiting the microbial community structure of the rice paddy rhizosphere but also shed light on uncovering environmental indicator microbes for transgenic *Bt* rice.

## Data Availability

The original contributions presented in the study are included in the article.

## Supplementary Materials

The following supporting information can be downloaded at: https://www.mdpi.com/article/10.3390/plants13101300/s1. Figure S1: Rarefaction analysis of TICs by treatments. Figure S2: Taxonomic structure at the phylum rank of the ACM (A) and TIC (B). Figure S3: Beta diversity of the TIC. Figure S4: Network analysis between samples. Figure S5: Rarefaction analysis of ACMs by treatments. Figure S6: Treatment-specific accumulations of ACM OTUs between GM and NonGM treatments. Figure S7: Microbiota comparisons of GM and NonGM samples from different sampling times. Figure S8: Microbiota comparison of CK and GM samples from different sampling times. Figure S9: Microbiota comparison of CK and NonGM samples from different sampling times. Figure S10: PCoA analysis of ACM samples by sampling times for each treatment based on Bray–Curtis distances. Figure S11: Identification of forty core OTUs. Figure S12: Taxonomical profiles of forty core ACM root communities. Figure S13: Heat map of forty core ACM OTUs in different samples. Figure S14: PCoA analysis using forty core ACM OTUs based on Bray–Curtis distances. Figure S15: Heatmap of ACM OTUs. Figure S16: Fourteen rice-enriched OTUs. Figure S17: Sixteen CK-enriched OTUs. Figure S18: Seventeen GM OTUs of significant difference in abundance between stages I and II. Figure S19: Stage-specific change in six GM-enriched OTUs between stage I and stage II. Figure S20: Clustering of samples from different treatments using ten GM-enriched ACM OTUs. Figure S21: Non-parametric Spearman rank correlation of OTU abundances in three random GM samples and their mean correlation values (filled circles) are plotted as a function of progressive thresholds (1 to 20) for the minimal number of sequences per OTU in a sample. Figure S22: Non-parametric Spearman rank correlation of OTU abundances in three random NonGM samples and their mean correlation values (filled circles) are plotted as a function of progressive thresholds (1 to 20) for the minimal number of sequences per OTU in a sample. Figure S23: Non-parametric Spearman rank correlation of OTU abundances in three random CK samples and their mean correlation values (filled circles) are plotted as a function of progressive thresholds (1 to 20) for the minimal number of sequences per OTU in a sample. Table S1: Detail information of each sample. Table S2: Sequencing counts and description of all predicted OTUs. Table S3: Sequencing counts and description of 2549 predicted OTUs in TIC dataset. Table S4: Sequencing counts and description of 168 predicted OTUs in ACM dataset. Table S5: Summary of 16S rDNA pyrosequencing. Table S6: Statistical test results of ACM OTUs from different treatments. Table S7: Statistical test of GM ACM OTUs from different rice developmental stages. Table S8: Statistical test of NonGM ACM OTUs from different rice developmental stages. Table S9: Statistical test of CK ACM OTUs from different rice developmental stages. Table S10: BLAST results to RDP database of cloned sequences selected from DDGE experiment. Table S11: Soil chemical properties measured in different treatments and different sampling stages. Table S12: Experimental design for each sample. Refs. [11,17,26,30,31,34,35,36,37,38] were cited in SM file.
