# Supplementary material for: Bt-Modified Transgenic Rice May Shift the Composition and Diversity of Rhizosphere Microbiota
Source: Plants (Basel). 2024 May 8;13(10):1300. doi: 10.3390/plants13101300 (PMC11125220; doi:10.3390/plants13101300)
Supplement: Supplementary file 1 [file plants-13-01300-s001.zip › Supplementary Figures.pdf]

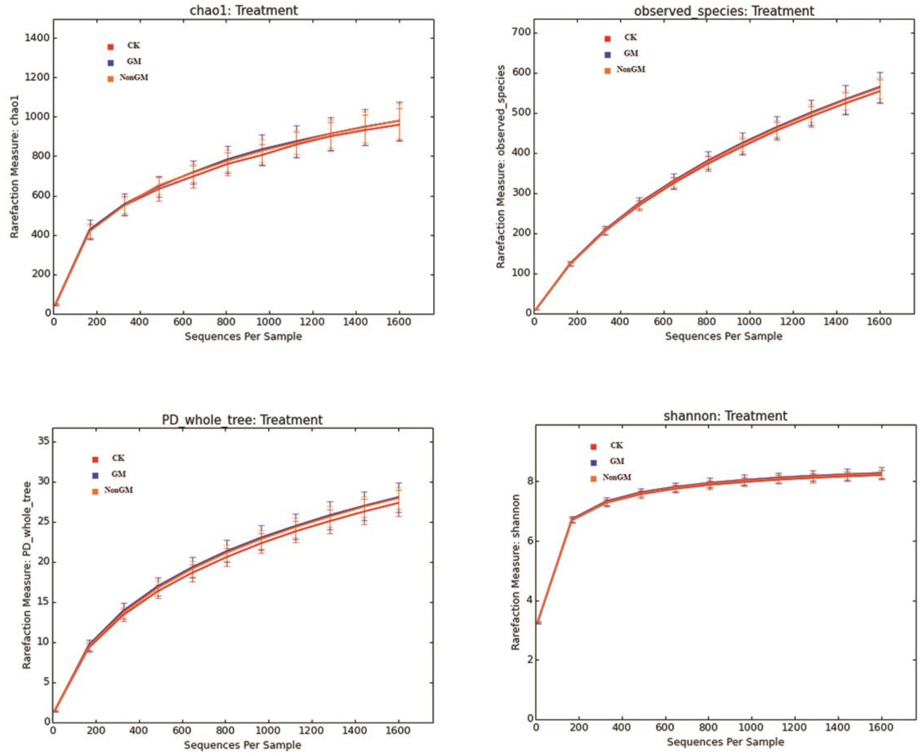

Fig. S1 Rarefaction analysis of TICs by treatments.

The TIC data matrix was used for multiple rarefactions (110 x tables from 100 -1,600 sequences per sample, steps of 160 sequences) by four different methods: Chao1, Observed species, PD whole tree, and Shannon.

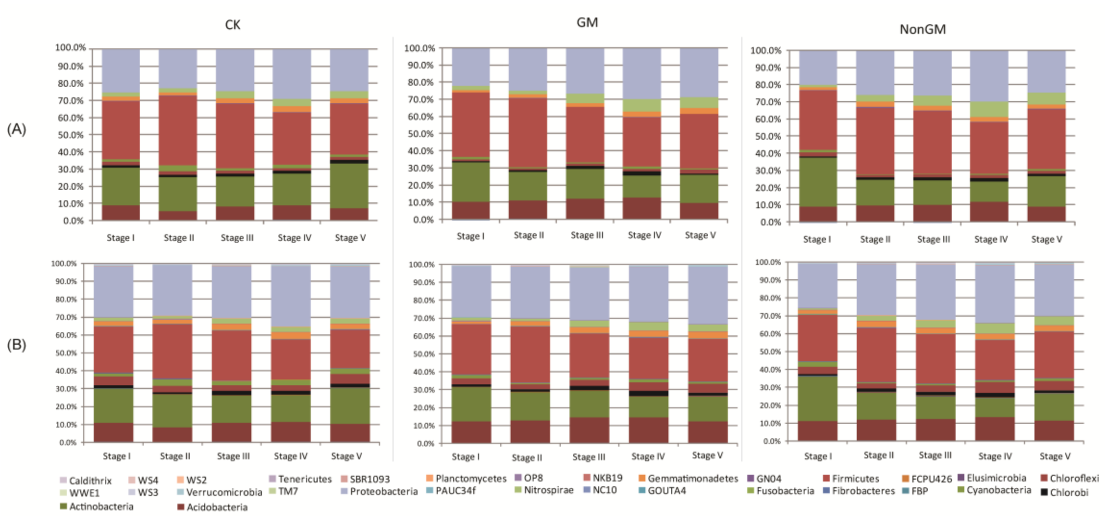

Fig. S2 Taxonomic structure at the phylum rank of the ACM (A) and TIC (B).

Data of 3 different treatments in 5 different sampling stages (Stage I to V) were shown. CK, blank control; GM, genetically modified rice; NonGM, non-transgenic rice.

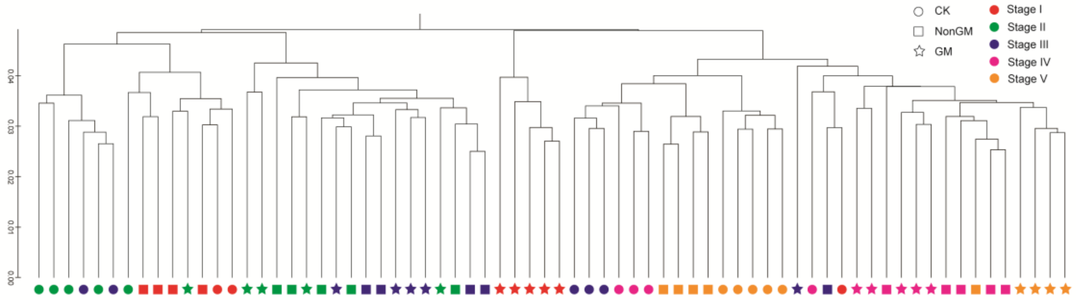

Fig. S3 Beta diversity of the TIC.

Between-sample diversity was calculated for TICs using weighted UniFrac distance metric (phylogeny-based and sensitive to the sequence abundances) on 1600 sequences per sample.

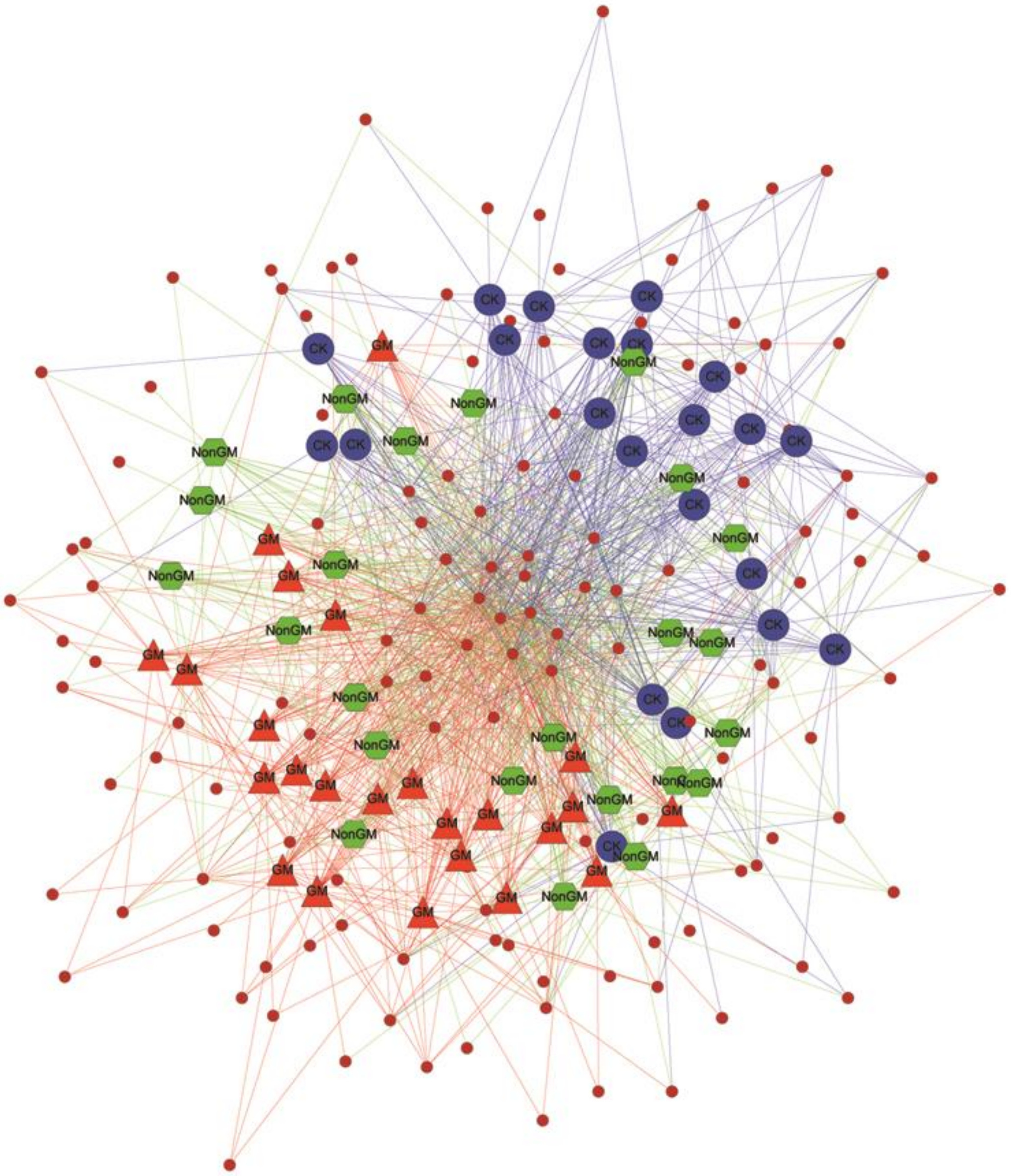

Fig. S4 Network analysis between samples.

Only ACM OTUs with relative abundance (RA) ⩾ 20 were shown. Red triangles, green hexagons, and blue circles stand for GM, NonGM, and CK samples, respectively; small red circles designated for ACM OTUs; lines connected with samples and OTUs mean OTUs present in corresponding samples.

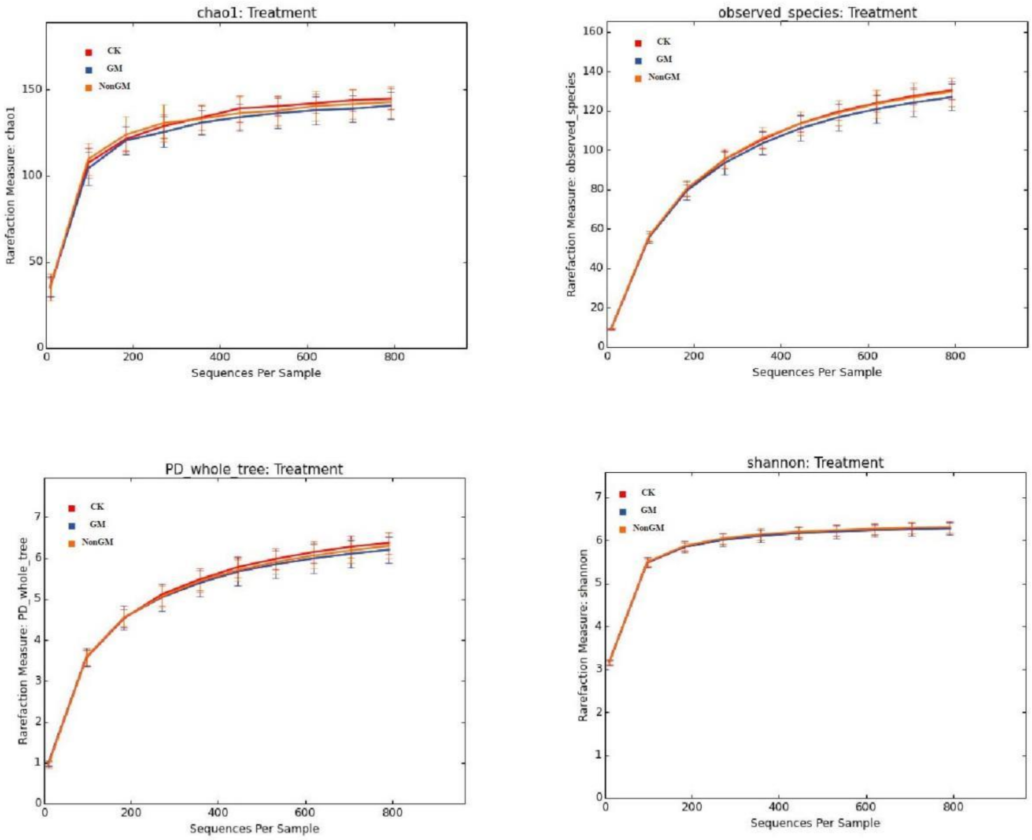

Fig. S5 Rarefaction analysis of ACMs by treatments.

The ACM data matrix was used for multiple rarefactions (110 x tables from 10 - 880 sequences per sample, steps of 87 sequences) by four different methods: Chao1, Observed species, PD whole tree, and Shannon.

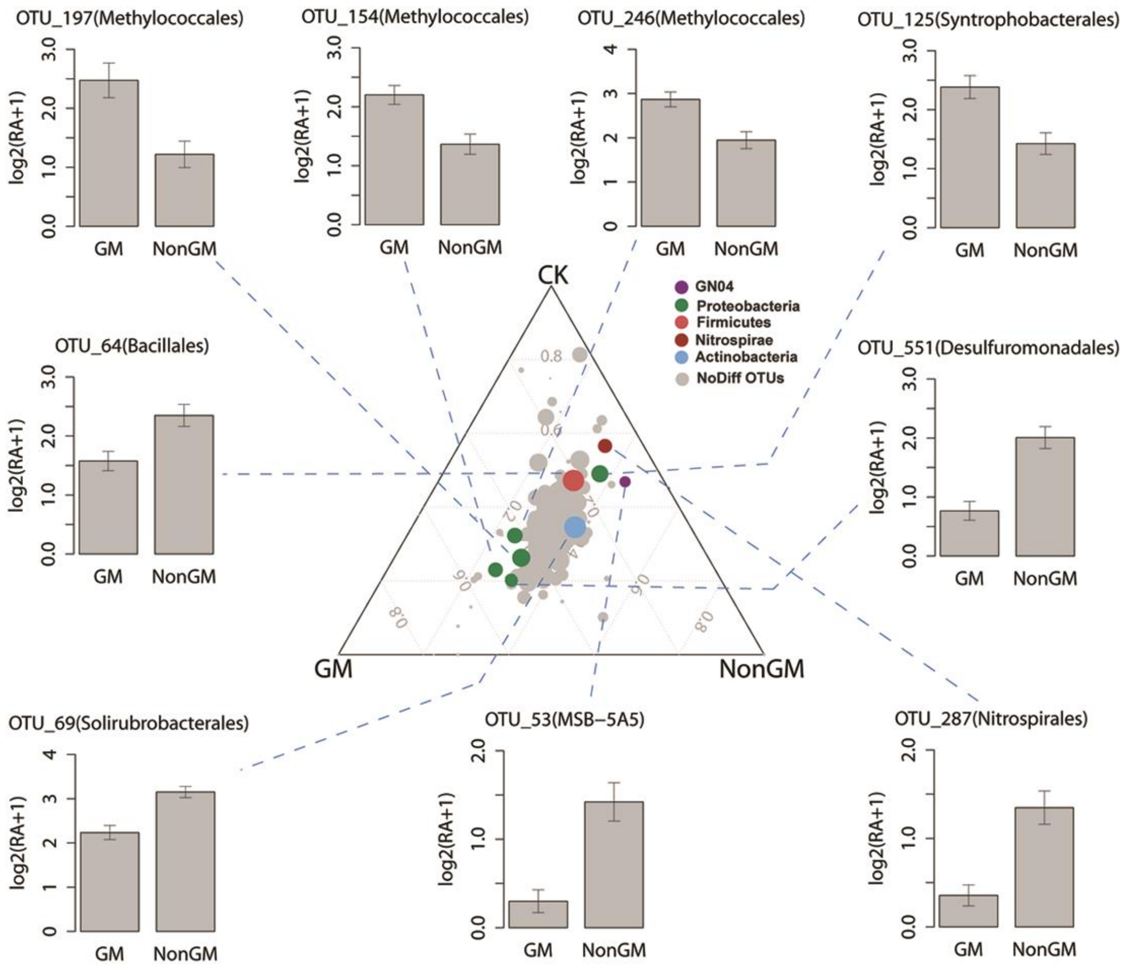

Fig. S6 Treatment-specific accumulations of ACM OTUs between GM and NonGM treatments. Relative abundance (RA) of all nine OTUs were significantly (FDR < 0.1) different between GM and NonGM rice.

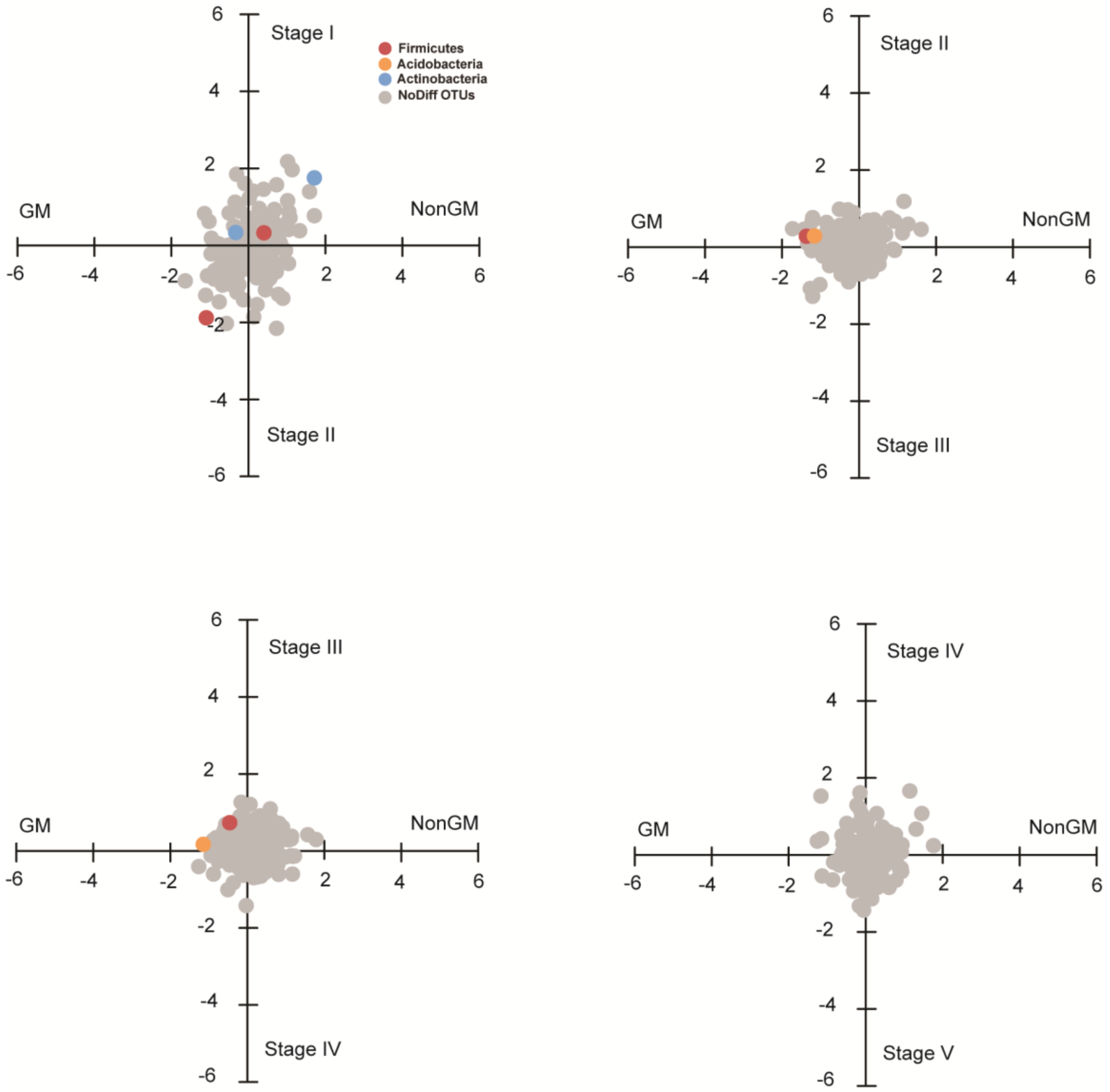

Fig. S7 Microbiota comparisons of GM and NonGM samples from different sampling times. Variation in mean relative abundance (RA) of individual OTUs (circles) across treatment and stages, where axes depict log2fold variation, x axis is log2(NonGM/ GM), y axis is log2(lower stage/ higher stage).

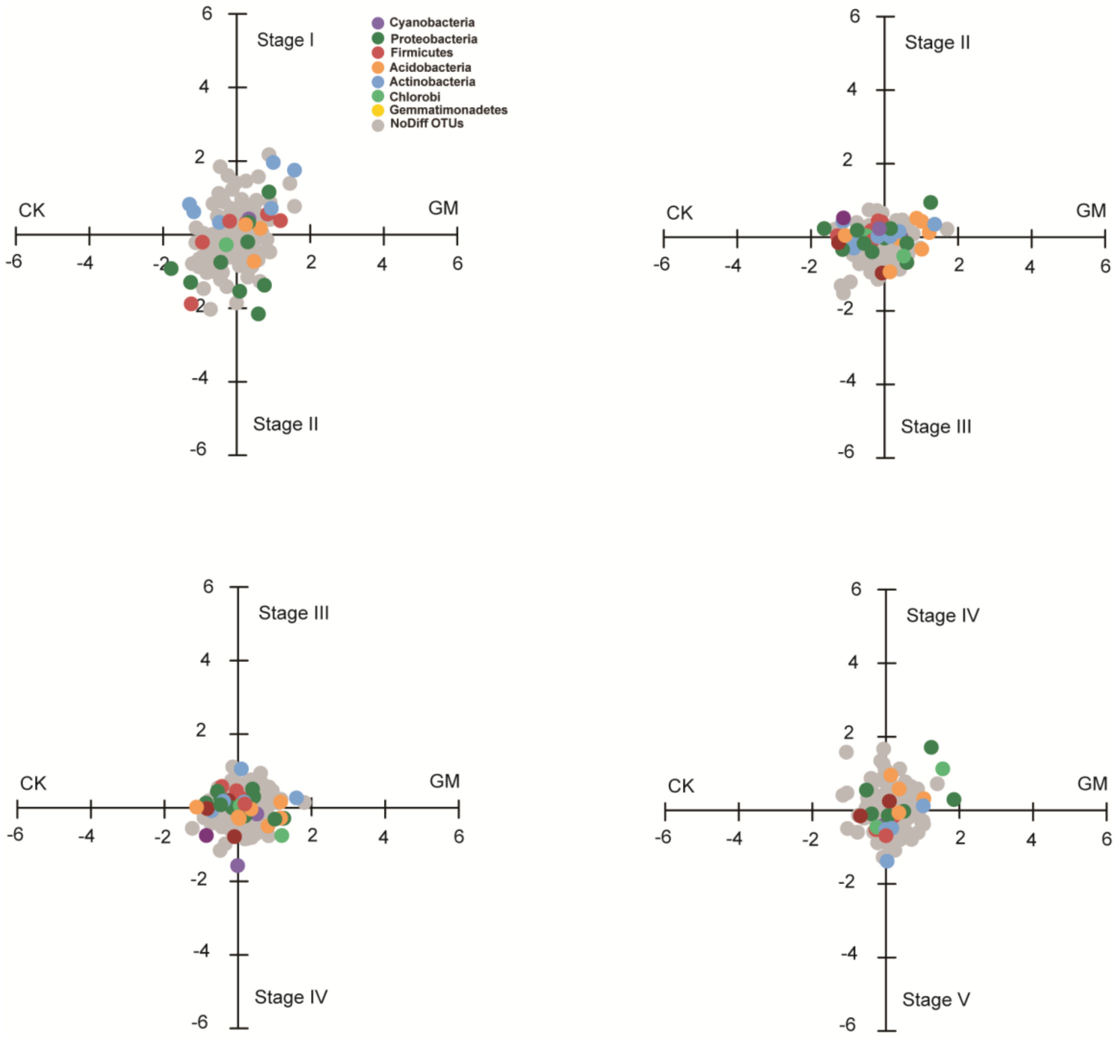

Fig. S8 Microbiota comparison of CK and GM samples from different sampling times.

Variation in mean relative abundance (RA) of individual OTUs (circles) across treatment and stages. Axes depicted log two-fold variation, x axis is log2(GM/CK), and y axis is log2(lower stage/higher stage).

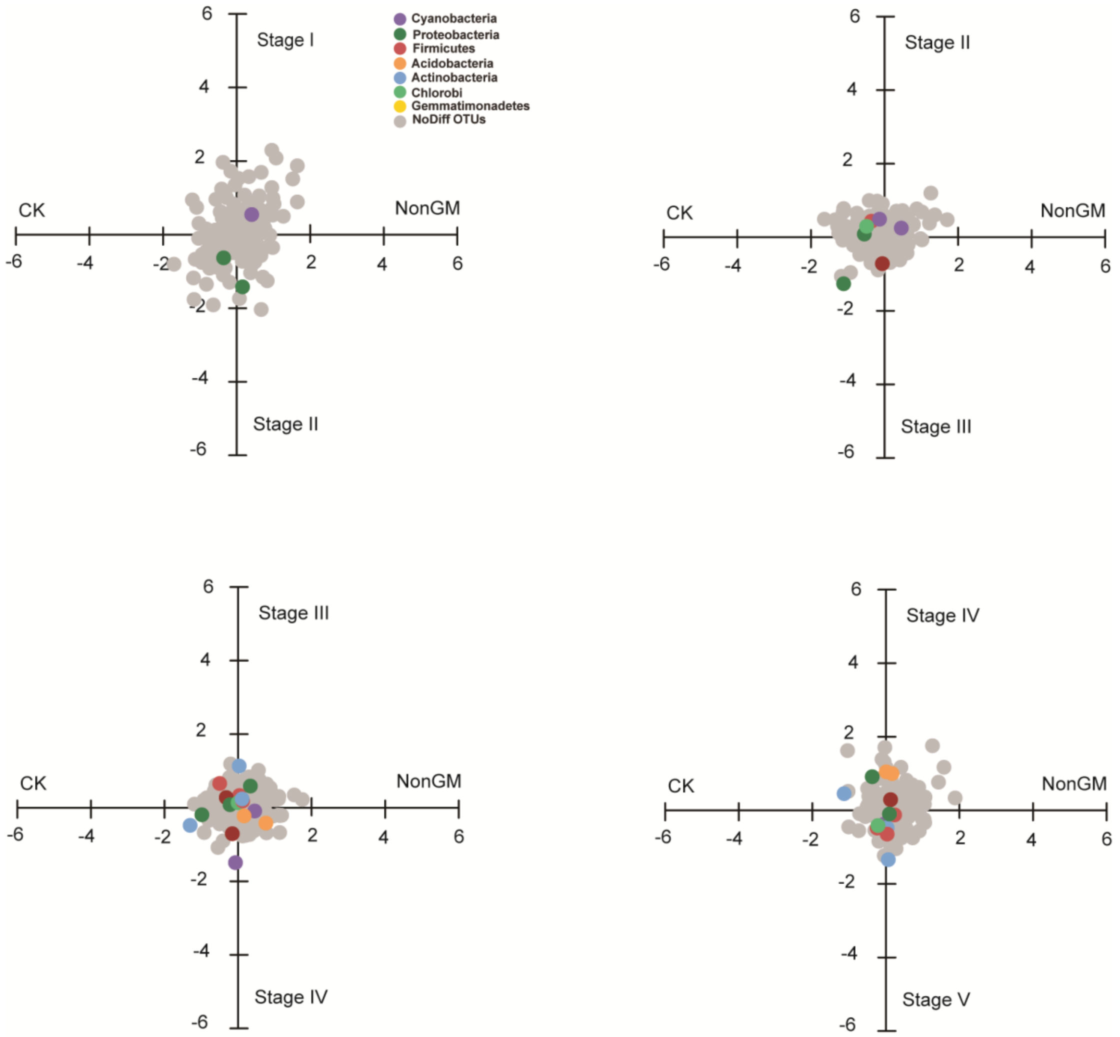

Fig. S9 Microbiota comparison of CK and NonGM samples from different sampling times.

Variation in mean relative abundance (RA) of individual OTUs (circles) across treatment and stages, where axes depicted log two-fold variation, x axis is log2(NonGM/CK), and y axis is log2(lower stage/higher stage).

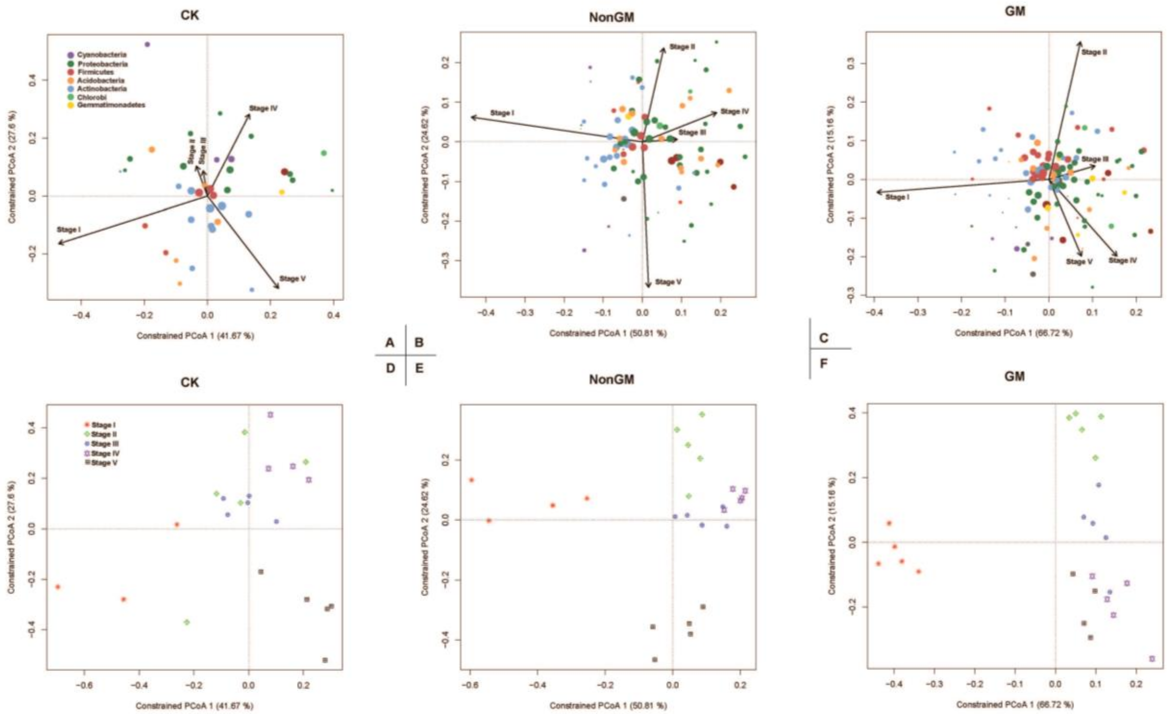

Fig. S10 PCoA analysis of ACM samples by sampling times for each treatment based on Bray-Curtis distances.

OTU scores of principle coordinate analysis of ACM OTUs for different treatment CK (A), NonGM (B) and GM (C) on Bray-Curtis distance were shown. Thesample scores of principle coordinate analysis of all samples for different treatment CK (D), NonGM (E) and GM (F) on Bray-Curtis distance were also shown. Scores were constrained by sampling times (stage I to stage V) and based on Bray-Curtis compositional dissimilarities. The arrows pointed to the centroid of the constrained factor. Circle size corresponds to relative abundance of OTUs/samples, and colors/shapes are assigned to different phyla/sample. The percentage of variation explained by each axis refers to the fraction of the total variance of the data explained by sampling times.

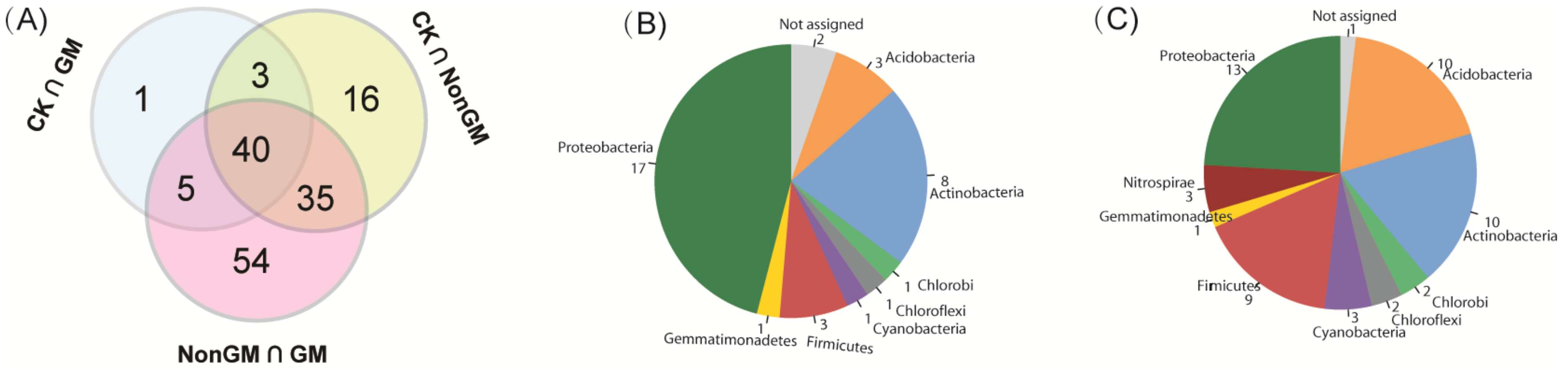

Fig. S11 Identification of 40 core OTUs.

Core OTUs were defined as OTUs shared among all treatments (FDR > 0.1) (A), taxonomic structure at the phylum rank for 40 core OTUs (B), and 54 GM and NonGM OTUs that were both significantly different to CK (FDR < 0.1) (C). CK ∩ GM, CK ∩ NonGM, and NonGM ∩ GM, designate for shared OTUs (FDR > 0.1) between two treatments.

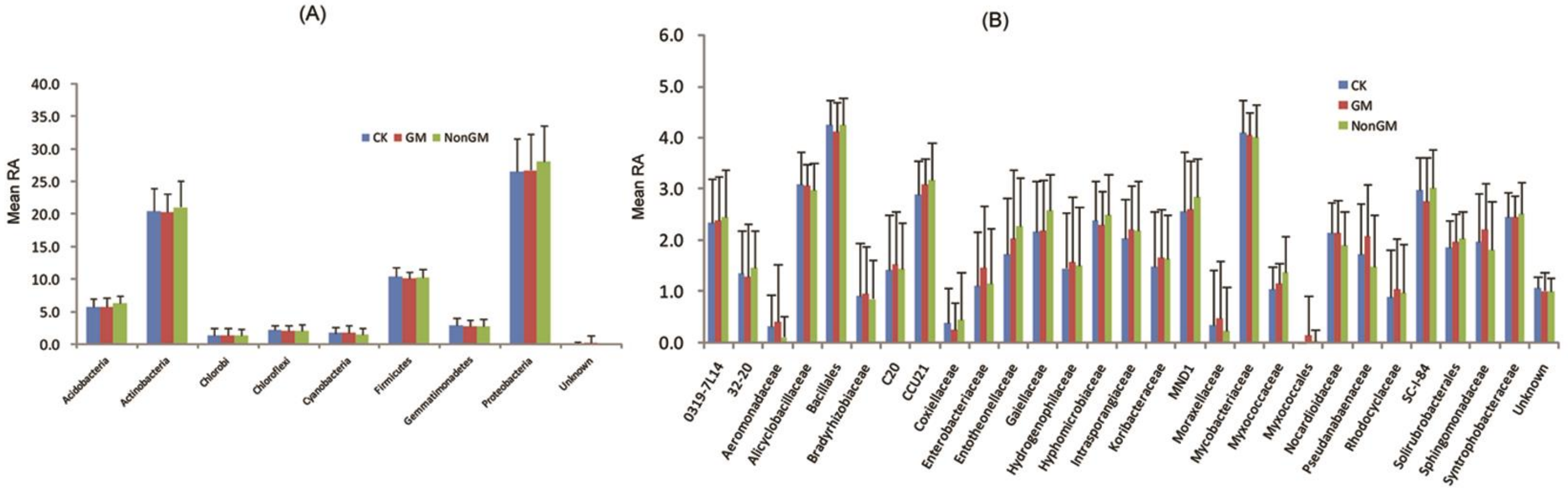

Fig. S12 Taxonomical profiles of forty core ACM root communities.

Mean relative abundance (RA, ± SEM) of taxa detected in root communities (color-coded by treatments) at the phylum (A), and the order/family rank (B) were shown. No significant differences (FDR > 0.1) were detected between CK, GM, and NonGM treatments.

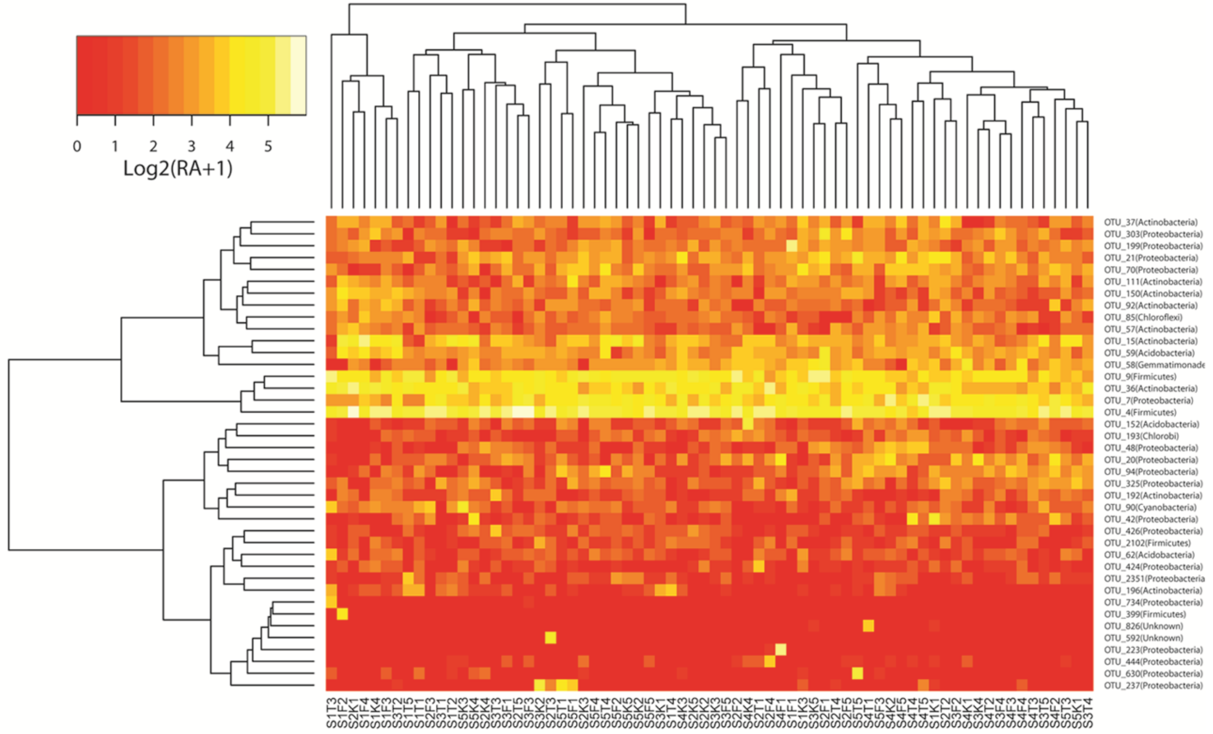

Fig. S13 Heat map of forty core ACM OTUs in different samples.

Samples from different treatment were basically of the same tendency in abundance change and could not be classified into different groups referring to different treatments.

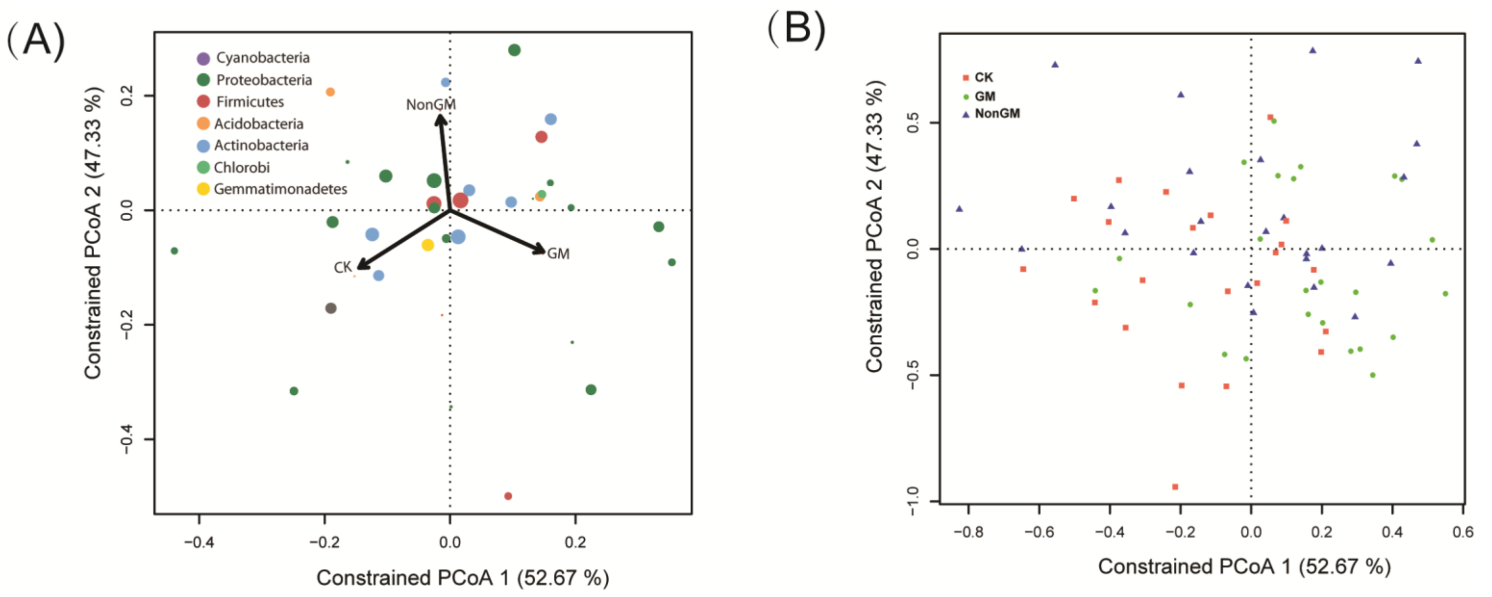

Fig. S14 PCoA analysis using 40 core ACM OTUs based on Bray-Curtis distances.

OTU scores of principle coordinate analysis of core ACM OTUs (A) and samples (B) from different treatment were shown. Scores were constrained by treatments and based on Bray-Curtis compositional dissimilarities. The arrows pointed to the centroid of the constrained factor. Circle size corresponds to relative abundance of OTUs/samples, and colors/shapes are assigned to different phyla/sample. The percentage of variation explained by each axis refers to the fraction of the total variance of the data explained by treatments.

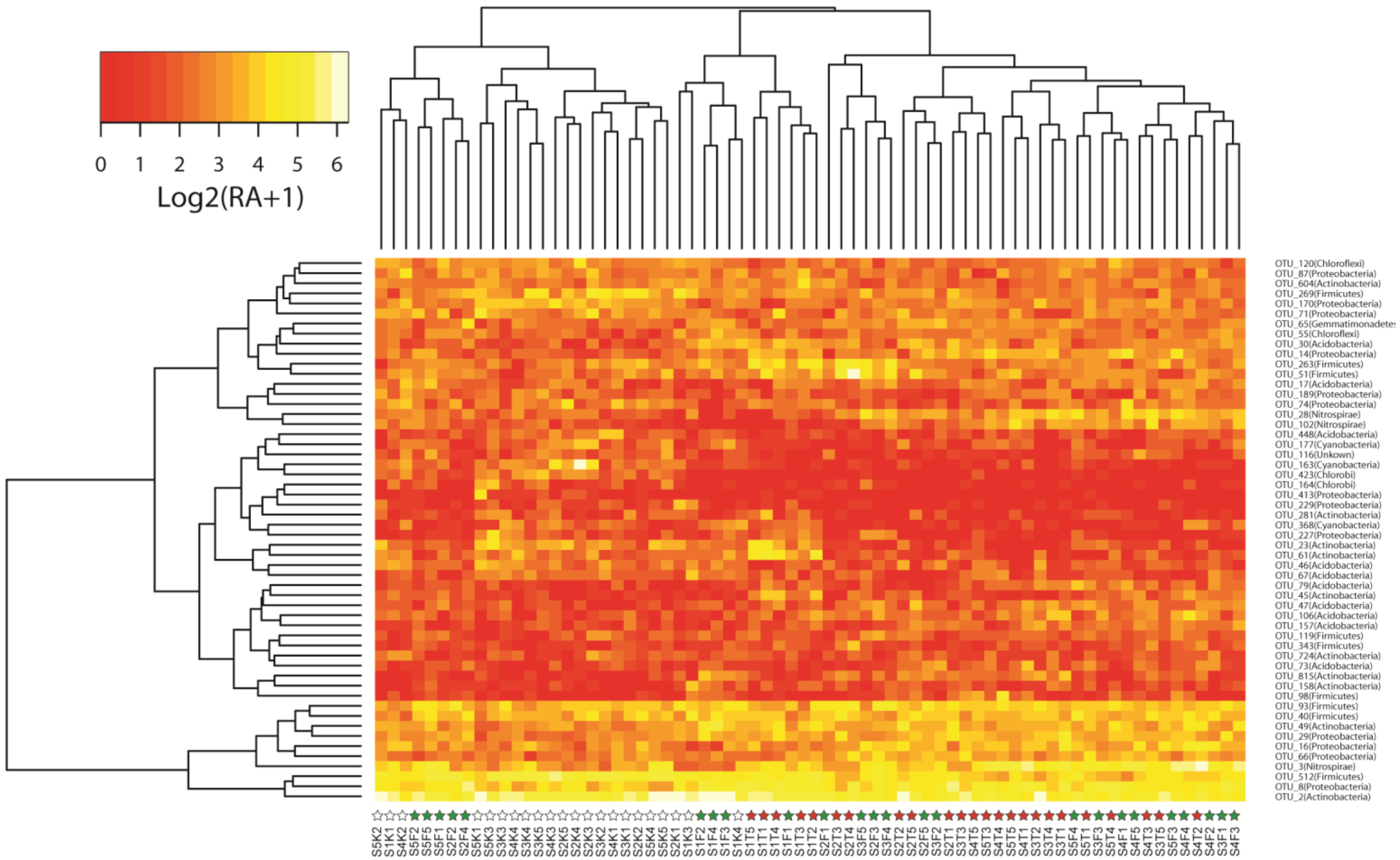

Fig. S15 Heatmap of ACM OTUs.

Fifty-four ACM OTUs of significant different abundance (FDR < 0.1) both in GM and NonGM treatments comparing with CK in different samples. Stars filled with colors of white, green, and red stand for CK, NonGM, and GM treatment samples, respectively; RA, relative abundance.

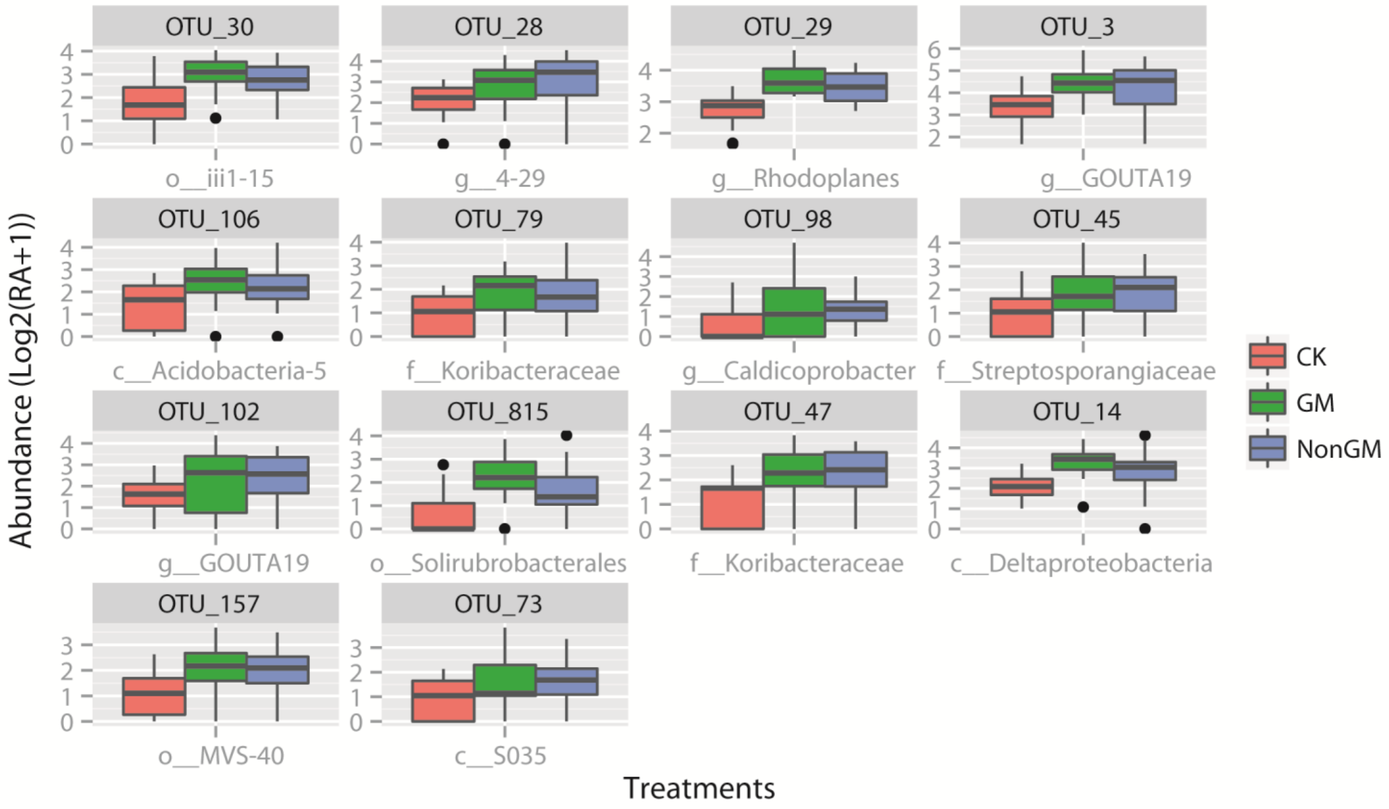

Fig. S16 Fourteen rice-enriched OTUs.

Rice-enriched OTUs were defined as OTUs of significant difference (FDR < 0.05) in abundance between GM and CK, and between NonGM and CK. Simultaneously, GM and NonGMOTU minimum mean abundance ⩾ 1 and the minimum up-regulated change fold compared to CK ⩾ 1.5-fold. Note: RA, relative abundance; o_, order; g_, genus; c_, class; f_, family.

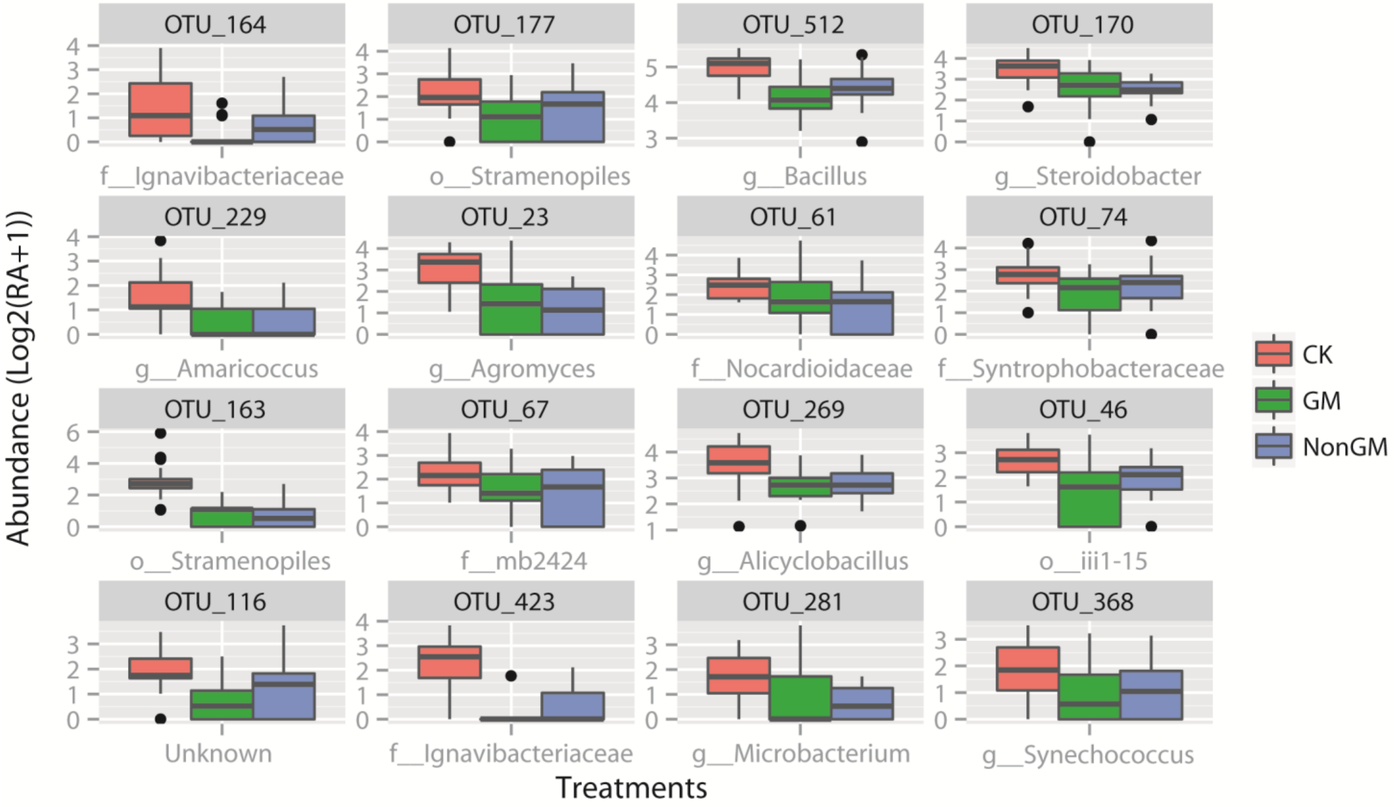

Fig. S17 Sixteen CK-enriched OTUs.

CK-enriched OTUs were defined as OTUs of significant difference (FDR < 0.05) in abundance between GM and CK, and between NonGMand CK. Minimum mean abundance log2(RA+1) of CK ⩾ 1. Comparing to GM and NonGM, the minimum up-regulated change fold of CK ⩾ 1.5-fold. RA, relative abundance; f_, family; o_, order; g_, genus; unknown, not assigned.

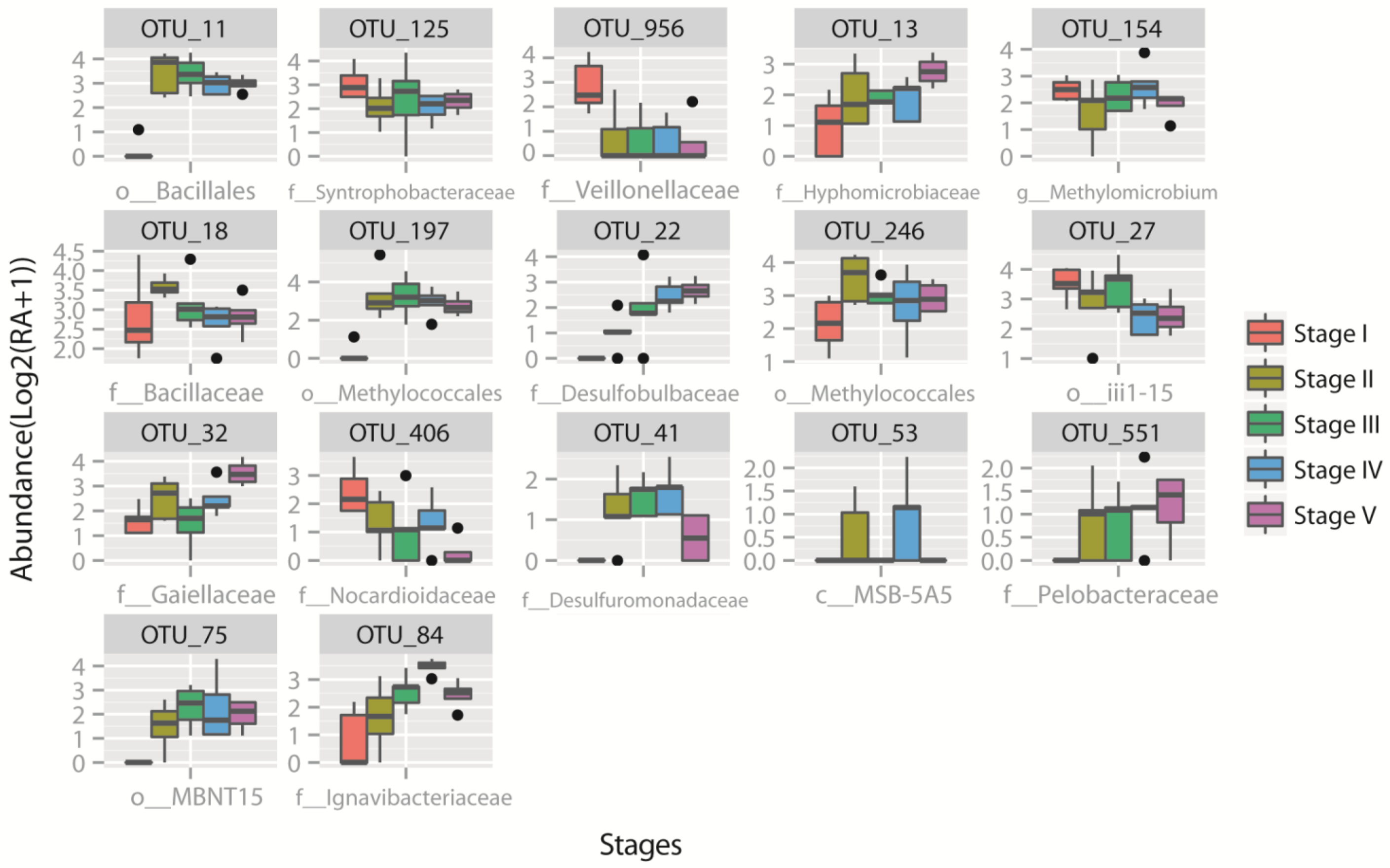

Fig. S18 Seventeen GM OTUs of significant difference in abundance between stages I and II.

Significant difference in abundance between stages I and II Minimum mean abundance (FDR < 0.1); log2(RA+1) of GM OTUs ⩾ 1; RA, relative abundance; f_, family; o_, order; g_, genus; c_, class.

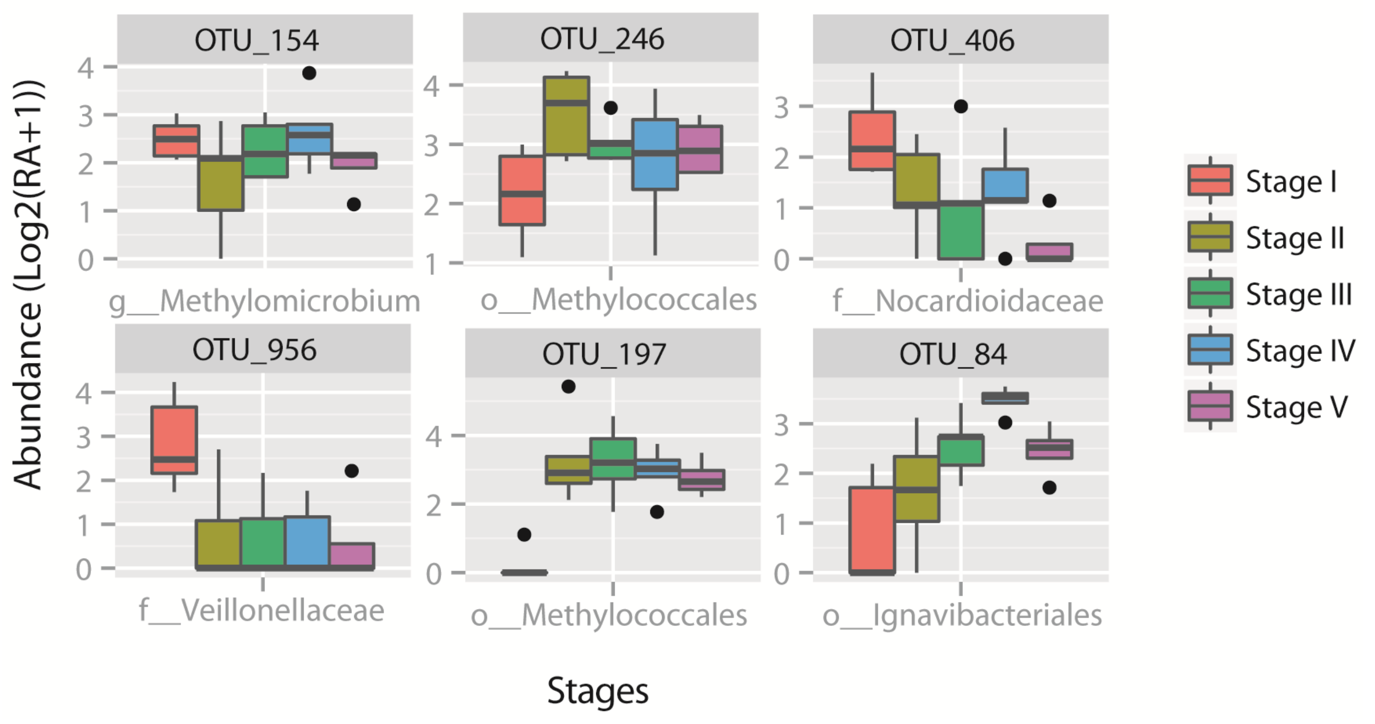

Fig. S19 Stage-specific change of six GM-enriched OTUs between stage I and stage II.
Abundances of these OTUs were significantly (FDR < 0.05) changed between stages I and II. RA, relative abundance; f_, family; o_, order; g_, genus.

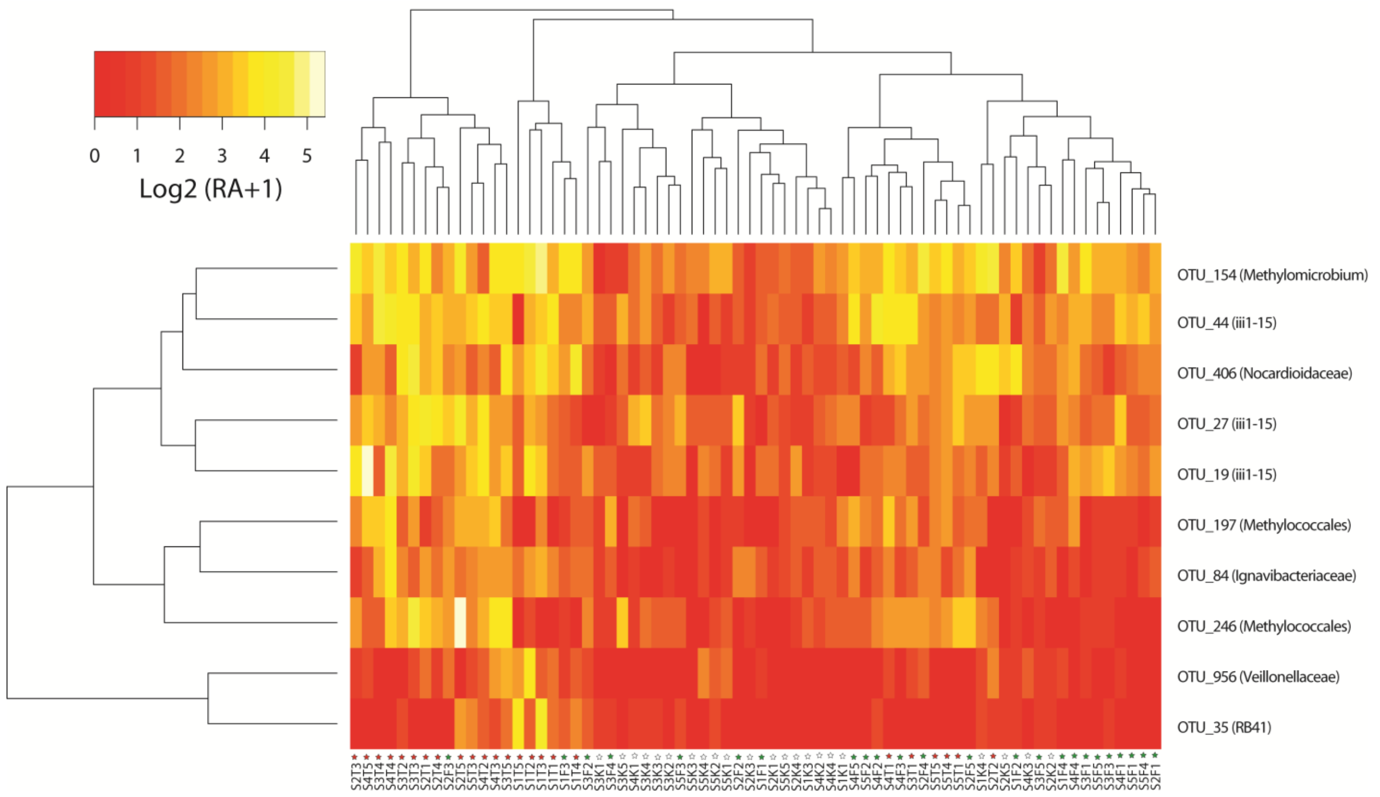

Fig. S20 Clustering of samples from different treatments using ten GM-enriched ACM OTUs.

Most of GM samples can be separated from CK and NonGM samples. Stars filled with colors of white, green, and red stand for CK, NonGM, and GM treatment samples, respectively; RA, relative abundance.

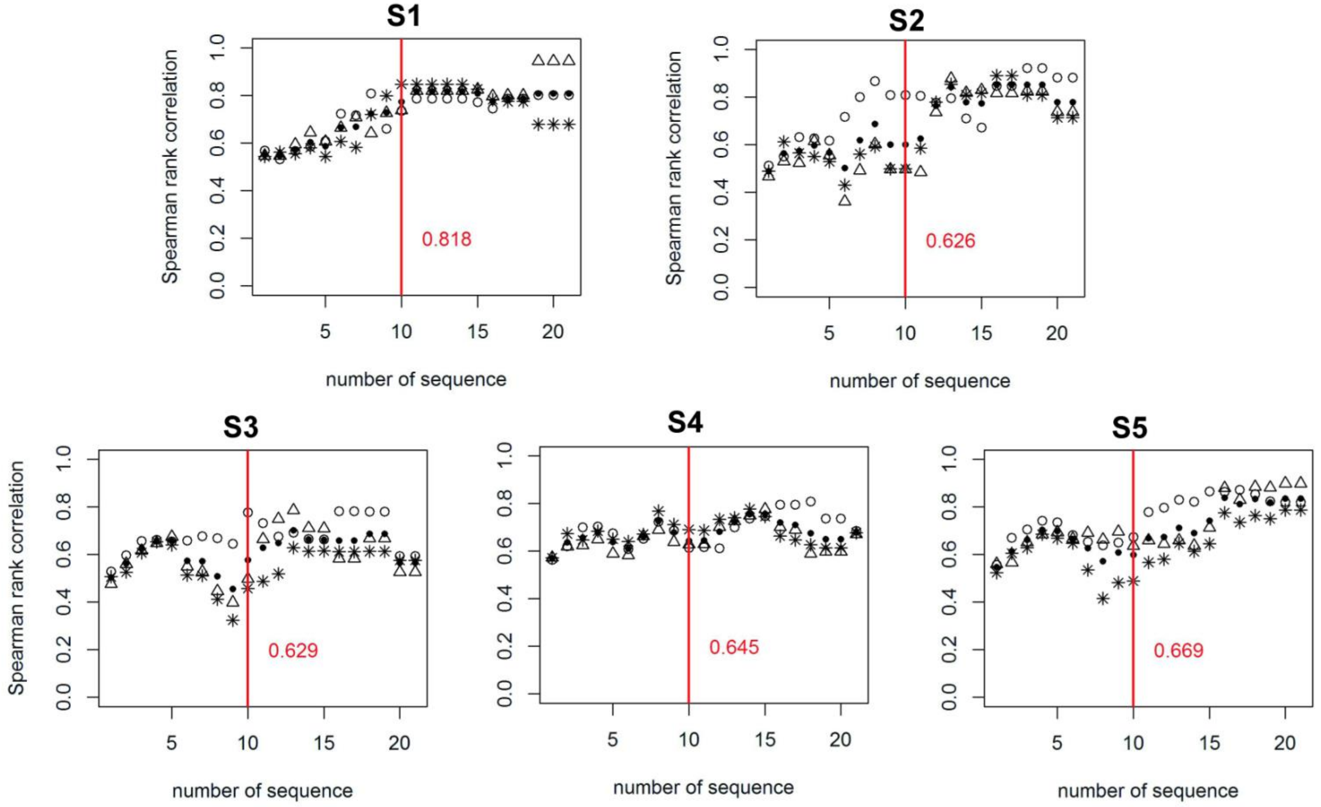

Fig. S21 Non-parametric Spearman rank correlation of OTU abundances in three random GM samples and their mean correlation values (filled circles) are plotted as a function of progressive thresholds (1 to 20) for the minimal number of sequences per OTU in a sample.

The red line indicates the threshold of 10 sequences per OTU and the corresponding Spearman rank correlation value is given in the plots. S1, Stage I; S2, Stage II; S3, Stage III; S4, Stage IV; S5, Stage V.

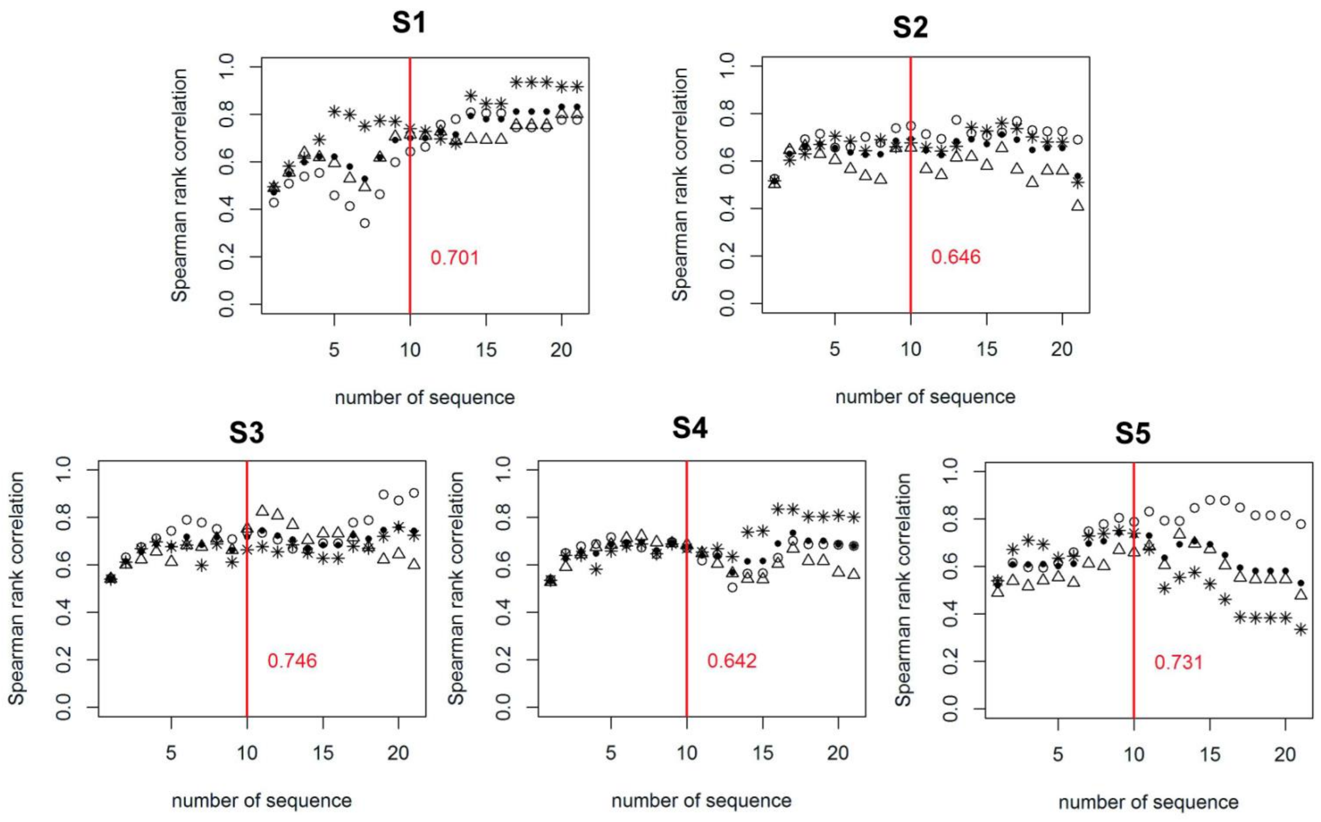

Fig. S22 Non-parametric Spearman rank correlation of OTU abundances in three random NonGM samples and their mean correlation values (filled circles) are plotted as a function of progressive thresholds (1 to 20) for the minimal number of sequences per OTU in a sample.

The red line indicates the threshold of 10 sequences per OTU and the corresponding Spearman rank correlation value is given in the plots. S1, Stage I; S2, Stage II; S3, Stage III; S4, Stage IV; S5, Stage V.

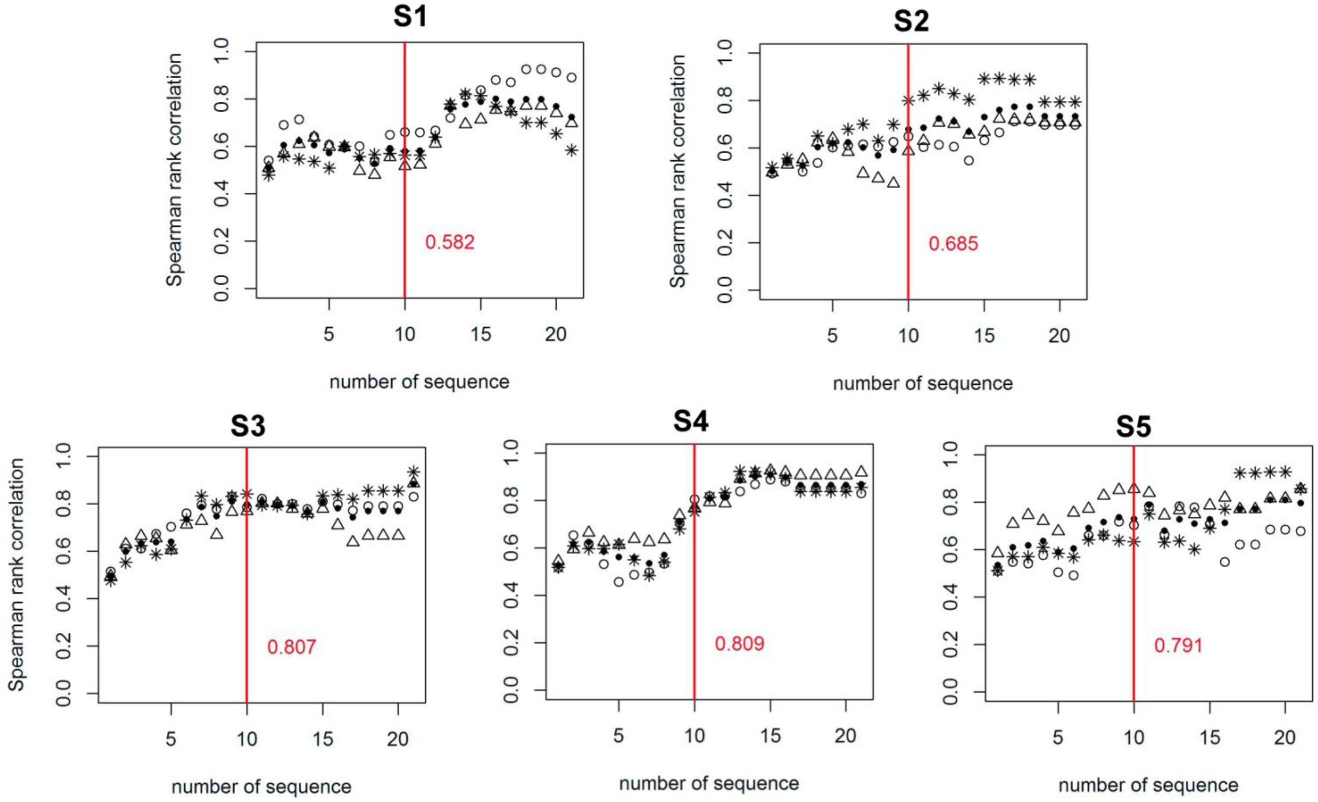

Fig. S23 Non-parametric Spearman rank correlation of OTU abundances in three random CK samples and their mean correlation values (filled circles) are plotted as a function of progressive thresholds (1 to 20) for the minimal number of sequences per OTU in a sample.

The red line indicates the threshold of 10 sequences per OTU and the corresponding Spearman rank correlation value is given in the plots. S1, Stage I; S2, Stage II; S3, Stage III; S4, Stage IV; S5, Stage V.
