# Supplementary material for: Bt-Modified Transgenic Rice May Shift the Composition and Diversity of Rhizosphere Microbiota
Source: Plants (Basel). 2024 May 8;13(10):1300. doi: 10.3390/plants13101300 (PMC11125220; doi:10.3390/plants13101300)
Supplement: Supplementary file 1 [file plants-13-01300-s001.zip › Supplementary material and method.pdf]

Supplementary information

Bt-modified transgenic rice may shift the composition and diversity of rhizosphere microbiota

Qixing Huang [1,2,3#], Yuliang Zhang [1,2,3#], Yanhua Tan[1,2,3], Hua Kong[1,2,3], Yang Cao[1,2,3], Jungang Wang[1,2], Guohua Yin [4,5*], Anping Guo [1,2,3*],

Materials and Methods

Plant materials

The Huahui No.1 transgenic *Bt* rice (event TT51-1) harboring *cry1Ab/Ac* gene was generated from its non-transgenic counterpart (Minghui63) by Huazhong Agricultural University [1]. Both rice lines were cultivated in the adjoining experimental paddy fields belonging to Sanya Science & Technology Academy for Crop Winter Multiplication (18°18′10″N/109°24′44″E, at an altitude of 27 meters, Sanya, Hainan, China) with identical environmental conditions.

Experimental design

Base fertilizer was applied to the experimental paddy field twenty days before rice seedlings were planted. No pesticides, antiseptics or fertilizers were applied thereafter. Field tests included three different treatments: paddy fields with Huahui No.1 rice (denoted as 'GM'), paddy fields with Minghui63 rice (denoted as 'NonGM') and paddy fields with no rice planted (denoted as 'CK'). Twenty days after seed germination, seedlings were transplanted to the experimental paddy fields. The field occupied dimensions of 28.5 m ×15.5 m (441.75 m^2) and was equally divided into 48 experimental plots. Each plot occupied an area of 3 m × 2 m (6 m^2) and was separated from other blocks by a 0.5 m space. One treatment was randomly applied to each plot, resulting in 16 replicates per treatment. Normal rice-farming practices were employed during the

growing season.

Sampling times were designated according to four different rice developmental stages after transplanting to paddy fields: seedling (18 days), tillering (61 days), panicle development (91 days) and maturation (119 days). Matured rice crop was harvested manually using sickles. Rice stubbles approximately 10 - 20 cm long were left in the fields. To further investigate the changes of rice rhizosphere microbes in the actual situation of farming, a post-harvest sampling time (145 days) was added, resulting in a total of five sampling times (denoted as stage I, stage II, stage III, stage IV and stage V, respectively). A detailed experimental design with individual sample IDs and sampling time is shown in Supplementary Table S12.

Sample preparation

In this study, each treatment consisted of 16 plots distributed in a field layout. Then, 5 out of 16 plots were randomly selected for sampling per treatment. Using the traditional five-point sampling method, we collected and pooled five rhizosphere soil samples per plot, generating 5 sample points per treatment. Each treatment was sampled at 5 different time points of plant growth stages (Stages I to V). A sterilized shovel was used to dig out the entire rice plants complete with roots and surrounding soil (approximately 5 cm). Soils within the range of 0.5 cm to 3 cm beneath the root base were removed. The roots were separated from the adhering soil particles upon harvest. Root-associated soils were collected in a 15 mL Eppendorf centrifugal tube containing 3 ml PBS-S buffer (130 mM NaCl, 7 mM Na_2HPO_4, 3 mM NaH_2PO_4, pH 7.0, 0.02% Silwet L-77) and washed for 20 minutes at 180 rpm on a shaking platform. The soil suspension was centrifuged for 20 minutes at 4 000 x g. The pellet, referred to as the rhizosphere, was collected in liquid nitrogen and stored at -80°C. Soil samples were collected from control plots in a soil depth of -3.0 to -6.0 cm from the surface corresponding to the approximate root depth in other plots, frozen in liquid nitrogen and stored at -80°C for further processing.

DNA extraction

DNA was extracted using the Fast DNA SPIN Kit for Soil (MP Biomedicals, Solon, Ohio, USA) following the manufacturer's instructions. DNA samples were eluted in 50 μL water. The quality, purity and quantity of the extracted DNA were assessed using a PGENERAL T6 Spectrophotometer (Beijing Purkinje General Instrument, Beijing, China). DNAs extracted from all samples were dissolved in 50 μL TE buffer, respectively. To evaluate the integrity and compare the productivity of the DNA, gel electrophoresis was carried out by loading 2 μL DNA solution and 8 μL H_2O on a 1% agarose gel (Life Technologies, Carlsbad, California, USA), stained with Goldview (Beijing SBS Genetech, Beijing, China), and visualized under UV light with the EC3 Imaging system (UVP, Upland, California, USA).

Barcode PCR and 16S rDNApyrosequencing

For pyrosequencing analysis, the extracted microbiome DNA concentration was further quantified using the Invitrogen Qubit 2.0 Fluorometer (Life Technologies) to ensure equal DNA concentration in PCR reactions. Barcode sequence tags were specifically designed for each sample (Supplementary Table S12). The V6-V8 regions of the 16S rDNA gene were amplified from soil DNA with the primer pair V6F3: 5'-CGTATCGCCTCCCTCGCGCCATCAG(barcode)TGCAACGCGAAGAACCTTACC-3' and V8R2: 5'-CTATGCGCCTTGCCAGCCCGCTCAGGCCCGGGAACGTATTCACCG-3' designed by Sangon Biotech, Shanghai, China. PCR was performed in a 50 μL volume system: 5 μL 10X Taq PCR Master Mix (TransGene, Beijing, China), 0.5 μL 50 pm/μL of each primer, 0.5 μL 10 μm/μL of dNTP, 0.5 μL 5 U/μL of Platinum Taq DNA polymerase, 1 μL 10 ng/μL genomic DNA, and enough sterilized H_2O to equate a volume of 50 μL. The following thermocycling program was used for PCR: (94°Cfor 3 min) × 1 cycle; (30 s at 94°C, 20 s at 45°C, 30 s at 65°C) × 5 cycles; (20 s at 94°C, 20 s at 55°C, 30 s at 72°C) × 20 cycles; and (72°C for 5 min) × 1 cycle, using Thermal Cycler Dice (TaKaRa Bio, Otsu, Japan). PCR products were purified using a SK8131 purification kit (Sangon Biotech, Shanghai, China). PCR product integrity was examined by standard 1.5% (w/v) agarose

1× TAE gel electrophoresis with SYBR Green I staining. DNA concentrations of PCR products were measured with Quant-it DNA BR (Life Technologies) according to the manufacturer's instructions. Then, the DNA was precipitated with ethanol and resuspended in water to approximately 1 μg/μL. All metagenomic libraries consisted of approximately 5 μg DNA. Sequencing was performed using the Genome Sequencer-FLX system (454 Life Sciences, Brandford Connecticut, USA) by Sangon Biotech. A summary of 16S rDNA pyrosequencing was listed in Supplementary Table S5.

Operational taxonomic unit prediction using UPARSE

Pyrosequencing reads were processed and analyzed using USEARCH (version 8.0.1477) [2]. Reads with erroneous barcodes, forward primer sequences or ambiguous base calls were discarded using the script fastq_strip_barcode_relabel2.py. The requirements for sequences to be considered 'quality' included a maximum expected truncation error of 0.5 (-fastq_maxee 0.5) and a length of at least 375 bp (-fastq_trunclen 375). As a result, 308,571 reads (67.7% of total) were admitted as quality sequences. These were indexed by samples and libraries into a single fasta file, which were further used for sequence de-replication analysis. 156,046 reads were selected as de-noised reads. De-noised reads were further sorted and used for singleton filtering analysis. Small reads with a count size of 3 or less (-minsize 3) were recognized as singletons and were discarded. As a result, 12,936 reads were selected as input for OTU predictions by the UPARSE algorithm [3]. Initial predictions estimated to find 2,689 OTUs and 2,478 chimeric sequences (19.2%) from the 12,936 reads. However, after filtration using the Greengenes 16S rRNA alignment reference database (release gg_13_8_99, rRNA16S.gold.NAST_ALIGNED.fasta) by USEARCH (-uchime_ref), 94 chimeras were found (3.5%), retrieving 2,595 non-chimeras. The qualified sequences at 97% sequence similarity defining the OTUs were mapped back to the OTUs by USEARCH (-usearch_global). Finally, the uc2otutab.py script was used to create an OTU table. Samples were rarefied to 1,600 sequences in each sample to correct differences of

sequencing depth.

Sequence analysis using QIIME

Sequence analysis steps were applied as described by Schlaeppi *et al.* [4] with some minor adjustments. The OTU table was converted into Biom format and taxonomically assigned with Greengenes (release gg_13_8_99) using QIIME (version 1.9.1) [5]. OTUs assigned to be plant chloroplast and mitochondria were identified manually using 'Streptophyta' and 'mitochondria' keywords. One OTU (OTU_930) was removed from the OTU table with the script filter_otus_from_otu_table.py. The resulting OTU table (S2Table) contained 2,579 OTUs. Two samples (S1K2 and S5T2) containing less than 1,600 OTUs (259 and 654, respectively) were removed from downstream analyses with the script filter_samples_from_otu_table.py. For the phylogeny related analyses, a tree was built based on PyNAST alignment [6] and filtered a set of representative sequences. The script multiple_rarefactions.py with the OTU table (Supplementary Table S2) was used as input to prepare the rarefied OTU tables (100 x tables between 1,000 to 1,600 sequences per sample with sets of 200 sequences), which were used for alpha diversity analyses (alpha_diversity.py with metrics chao1, observed_species, shannon and PD_whole_tree). We randomly chose the data file #93 (TIC.2549 seq.fa) of the rarefied OTU tables (sampling depth of 1,600 sequences per sample) for the downstream OTU-based and beta diversity analyses. We referred to this data matrix as TIC for which the taxonomic overview was presented in Fig. S1B. Supplementary table S3 reported the number of quality sequences per OUT in each sample in the TIC data matrix. The beta diversity was estimated by calculating weighted UniFracdistances with the script beta_diversity.py. The distance matrix was imported into R (version 3.1.2) to generate the Supplementary Fig. S3 [7].

Defining the Abundant Community Members (ACMs)

ACMs were defined and analyzed following analyses methods as reported by Schlaeppi *et al.* [4]. In each treatment of the same sampling time (supplementary Table S2),

we tested non-parametric Spearman rank correlation of OTU abundances in three random samples and their mean correlation values between each two samples. Each OUT was represented from the minimal number of 1 to 20 sequences in a sample (supplementary Fig. S21, S22, S23). ACMs data matrices (Supplementary Dataset S2) were prepared by removing OTUs, which did not reach the minimum of 10 quality sequences in at least one of the 70 samples of all experiments. The counts of individual ACM OTUs were normalized in a sample by dividing the total counts of all ACM OTUs within that sample followed by a multiplication by 1,000 resulting in relative abundance (RA) expressed as per million. Statistical comparisons were conducted on log_2-transformed (RA+1) per million values. Supplementary Table S4 lists the number of quality sequences per OTU in each sample in the ACM data matrix and Supplementary Fig. S2A displays the taxonomic composition of the ACM. Alpha and beta diversity analyses for the ACM were performed in QIIME using the same functions and parameters as for the TIC analyses described above. To this end, the OTU-IDs of the ACM determined in R (version 3.1.2) were used in QIIME to subset the TIC data file (TIC.2549seq.fa) within the ACM data matrix (ACM.168seq.fa). The ACM data matrix was used for multiple rarefactions (110 x tables from 10 to 880 sequences per sample, steps of 87 sequences) to prepare the rarefied ACM containing OTU tables, which were subsequently employed for the ACM alpha diversity analyses This data was also imported into R (version 3.1.2) to generate Supplementary Fig. S5. Beta diversity of ACM was estimated based on 800 sequences per sample and the distance matrix was imported into R (version 3.1.2) to generate Fig. 1.

Statistical analysis

Data were analyzed statistically by one-way analysis of variance (ANOVA) and Tukey's test (HSD, threshold $P < 0.05$) using R (version 3.1.2).

Defining shared and core OTUs

'Core OTUs' were defined when they were supported by parametric Tukey (ANOVA), non-parametric Mann-Whitney and Bayesian statistics following analysis

methods as reported by Schlaeppi *et al*. [4]. The intersection of the three methods revealed 40 shared CoreOTUs for GM, NonGM, and CK experiments (Supplementary Fig. S11). Finally, the shared OTUs between the GM and NonGM experiments were compared and 14 core rice-enriched OTUs were revealed from their overlap (Supplementary Fig. S16).

Canonical analysis of principal coordinates

To assess the influence of the different experimental factors on beta diversity we calculated Bray-Curtis distances and performed a Canonical Analysis of Principal coordinates constrained by the factor of interest and conditioned by the remaining variables [8]. We employed R package version v2.2-1 for the constrained ordination ('capscale' function for CAP analysis) as well as for the calculation of the significance values and confidence intervals ('permutest' permutation-based testing function) [9].

ELISA test of *Bt* protein concentration levels

Along with the sampling of soil samples, we also collected rice leaves from Huahui No.1 transgenic *Bt* rice (event TT51-1) and its non-transgenic counterpart Minghui63 rice at four different rice developmental stages (stages I to IV). All collected rice leaves were ground into a fine powder in liquid nitrogen using a mortar and pestle, and then stored at -80°C for further study. The *Bt* toxin protein content in rice leaves was measured by the enzyme-linked immunosorbent assay (ELISA) using the Quantiplate Kit for Cry1Ab/Cry1Ac (Envirologix, Inc, USA), which was pre-coated with Cry1Ac antibody containing 96 well solid microplates. 1 mg of lyophilized powder of rice samples were homogenized in 1 mL of extraction buffer provided by manufacturer, then, were centrifuged at 12 000 rpm for 3 min. The immunoassay was performed according to the protocols provided by the manufacturer, in sequential steps, with a washing step that included a phosphate-buffered saline buffer (PBS with 0.05% Tween 20, pH = 7.4) in between the reagent addition. Samples and standards (calibrators) were added to the plates in triplicates and incubated at ambient temperature for 1 to 2 hours. Following the wash, horseradish peroxidases were utilized as a reporter enzyme and were conjugated

to Cry1Ac/Cry1Ab-specific antibodies, diluted in PBS phosphate-buffered saline buffer (PBS with 0.05% Tween 20, pH = 7.4) and added after 15 min of incubation. Lastly, 1.0 N hydrochloric acid was used to stop the reaction. Absorbance was measured at 450 nm using a Varioskan Flash Spectral Scan Multimode Plate Reader (Thermo Fisher Scientific, Waltham, Massachusetts, USA). A standard curve was established using cry1Ac standard protein at concentration ranged from 0.1 to 0.5 pg/mL.

DDGE analysis of 16S rDNAs in different samples

Microbiome DNA belonging to the same treatment at the sampling time from rhizosphere soils were pooled (n = 5). The V6 region of the 16S rDNA gene was amplified from soil DNA with the primer pair of F968 (5′CGCCCGCCGCGCGCGGCGGGCGGGGCGGGGGCACGGGGGGAACGCGAAGA ACCTTAC3′) and R1401 (5′CGGTGTGTACAAGACCC3′) introduced by Nubel and collegues [10]. PCR was performed in a 50 μL volume system: 5 μL 10× taq PCR Master Mix (TransGene, Beijing, China), 0.5 μL 50 pm/μL of each primer, 0.5 μL 10 μm/μL of dNTP, 0.5 μL 5 U/μL of Gibco Platinum Taq DNA polymerase, 1 μL 10 ng/μL genomic DNA, and 42 μL sterilized H_2O. The following thermocycling program was used for PCR: (94°C for 4 mins) × 1 cycle; (30 s at 94°C, 60 s at 56°C, 30 s at 72°C) × 30 cycles; and (420 s at 72°C) × 1 cycle, using Thermal Cycler Dice (TaKaRa Bio).Approximately 400 ng of PCR products were used for denaturing gradient gel electrophoresis (DGGE) analysis. Denaturant gels (30% and 60%) were made by mixing 0% and 100% denaturing stock solution containing 8% acrylamide, 150 μL 10% ammonium persulfate and 15 μL tetramethylethylenediamine with each solution. Electrophoresis was performed at a constant voltage of 180 V and a temperature of 60°C with 1× TAE buffer for 4 h using the DCode universal mutation system (Bio-Rad Laboratories, Hercules, California, USA). After electrophoresis, gels were stained with 5% Goldview for 30 mins, and the bands were visualized with a LumiVisonPRO 400EX (Taitec, Tokyo, Japan). Bands of interest were cut using sterilized blades and the DNA was obtained using SK1131 DNA gel

extraction kit. Then DNA was further cloned and sequenced by Sangon Biotech. Sequences were further analyzed according to the Ribosomal Database Project (RDP) using default parameters online (http://rdp.cme.msu.edu/seqmatch/index.jsp).

Measurement of soil chemical properties

To investigate the change of soil chemical properties, soil samples collected from experimental fields of different treatments in seedling stage (stage I) and the post-harvest sampling time (stage V) were used. Soil chemical properties including PH, organic carbon, total nitrogen, total phosphorus, total potassium, available nitrogen, available potassium and available phosphorus were measured by the Analysis and Testing Center, Chinese Academy of Tropical Agricultural Sciences.

References

1. Lu, C. The first approved transgenic rice in China. *GM Crops* **2010**, *1*, 113-115.
2. Edgar, R.C. Search and clustering orders of magnitude faster than BLAST. *Bioinformatics* **2010**, *26*, 2460-2461.
3. Edgar, R.C. UPARSE: highly accurate OTU sequences from microbial amplicon reads. *Nature Methods* **2013**, *10*, 996-998.
4. Schlaeppi, K.; Dombrowski, N.; Oter, R.G.; van Themaat, E.V.L.; Schulze-Lefert, P. Quantitative divergence of the bacterial root microbiota in *Arabidopsis thaliana* relatives. *Proceedings of the National Academy of Sciences* **2014**, *111*, 585-592.
5. Caporaso, J.G.; Kuczynski, J.; Stombaugh, J.; Bittinger, K.; Bushman, F.D.; Costello, E.K.; Fierer, N.; Pena, A.G.; Goodrich, J.K.; Gordon, J.I. QIIME allows analysis of high-throughput community sequencing data. *Nature Methods* **2010**, *7*, 335-336.
6. Caporaso, J.G.; Bittinger, K.; Bushman, F.D.; DeSantis, T.Z.; Andersen, G.L.; Knight, R. PyNAST: a flexible tool for aligning sequences to a template alignment. *Bioinformatics* **2010**, *26*, 266-267, doi:10.1093/bioinformatics/btp636.
7. Lozupone, C.; Knight, R. UniFrac: a new phylogenetic method for comparing microbial communities. *Applied and Environmental Microbiology* **2005**, *71*, 8228-8235.
8. Anderson, M.J.; Willis, T.J. Canonical analysis of principal coordinates: a useful method of constrained ordination for ecology. *Ecology* **2003**, *84*, 511-525.

9. Oksanen, J.; Blanchet, F.G.; Kindt, R.; Legendre, P.; Minchin, P.R.; O'Hara, R.; Simpson, G.L.; Solymos, P.; Stevens, M.; Wagner, H. vegan: community ecology package. R package version 2.0-10. 2013. **2015**.
10. Nübel, U.; Engelen, B.; Felske, A.; Snaidr, J.; Wieshuber, A.; Amann, R.I.; Ludwig, W.; Backhaus, H. Sequence heterogeneities of genes encoding 16S rRNAs in Paenibacillus polymyxa detected by temperature gradient gel electrophoresis. *Journal of bacteriology* **1996**, *178*, 5636-5643.
