# Supplementary material for: Bt-Modified Transgenic Rice May Shift the Composition and Diversity of Rhizosphere Microbiota
Source: Plants (Basel). 2024 May 8;13(10):1300. doi: 10.3390/plants13101300 (PMC11125220; doi:10.3390/plants13101300)
Supplement: Supplementary file 1 [file plants-13-01300-s001.zip › Table S1-S12/Table S12.docx]

**Table S12 Experimental design for each sample.**

| **Samples^*^** | **Treatments^#^** | **Sampling stages** | **Days after transplanting** | **Developmental stages** | **Samples** | **Treatments^#^** | **Sampling stages** | **Days after transplanting** | **Developmental stages** |
| --- | --- | --- | --- | --- | --- | --- | --- | --- | --- |
| S1F1 | NonGM | StageI | 18 | Seedling | S3K4 | CK | StageIII | 91 | Panicle development |
| S1F2 | NonGM | StageI | 18 | Seedling | S3K5 | CK | StageIII | 91 | Panicle development |
| S1F3 | NonGM | StageI | 18 | Seedling | S3T1 | GM | StageIII | 91 | Panicle development |
| S1F4 | NonGM | StageI | 18 | Seedling | S3T2 | GM | StageIII | 91 | Panicle development |
| S1K1 | CK | StageI | 18 | Seedling | S3T3 | GM | StageIII | 91 | Panicle development |
| S1K2 | CK | StageI | 18 | Seedling | S3T4 | GM | StageIII | 91 | Panicle development |
| S1K3 | CK | StageI | 18 | Seedling | S3T5 | GM | StageIII | 91 | Panicle development |
| S1K4 | CK | StageI | 18 | Seedling | S4F1 | NonGM | StageIV | 119 | Ripening |
| S1T1 | GM | StageI | 18 | Seedling | S4F2 | NonGM | StageIV | 119 | Ripening |
| S1T2 | GM | StageI | 18 | Seedling | S4F3 | NonGM | StageIV | 119 | Ripening |
| S1T3 | GM | StageI | 18 | Seedling | S4F4 | NonGM | StageIV | 119 | Ripening |
| S1T4 | GM | StageI | 18 | Seedling | S4F5 | NonGM | StageIV | 119 | Ripening |
| S1T5 | GM | StageI | 18 | Seedling | S4K1 | CK | StageIV | 119 | Ripening |
| S2F1 | NonGM | StageII | 61 | Tillering | S4K2 | CK | StageIV | 119 | Ripening |
| S2F2 | NonGM | StageII | 61 | Tillering | S4K3 | CK | StageIV | 119 | Ripening |
| S2F3 | NonGM | StageII | 61 | Tillering | S4K4 | CK | StageIV | 119 | Ripening |
| S2F4 | NonGM | StageII | 61 | Tillering | S4T1 | GM | StageIV | 119 | Ripening |
| S2F5 | NonGM | StageII | 61 | Tillering | S4T2 | GM | StageIV | 119 | Ripening |
| S2K1 | CK | StageII | 61 | Tillering | S4T3 | GM | StageIV | 119 | Ripening |
| S2K2 | CK | StageII | 61 | Tillering | S4T4 | GM | StageIV | 119 | Ripening |
| S2K3 | CK | StageII | 61 | Tillering | S4T5 | GM | StageIV | 119 | Ripening |
| S2K4 | CK | StageII | 61 | Tillering | S5F1 | NonGM | StageV | 145 | Post-harvest |
| S2K5 | CK | StageII | 61 | Tillering | S5F2 | NonGM | StageV | 145 | Post-harvest |
| S2T1 | GM | StageII | 61 | Tillering | S5F3 | NonGM | StageV | 145 | Post-harvest |
| S2T2 | GM | StageII | 61 | Tillering | S5F4 | NonGM | StageV | 145 | Post-harvest |
| S2T3 | GM | StageII | 61 | Tillering | S5F5 | NonGM | StageV | 145 | Post-harvest |
| S2T4 | GM | StageII | 61 | Tillering | S5K1 | CK | StageV | 145 | Post-harvest |
| S2T5 | GM | StageII | 61 | Tillering | S5K2 | CK | StageV | 145 | Post-harvest |
| S3F1 | NonGM | StageIII | 91 | Panicle development | S5K3 | CK | StageV | 145 | Post-harvest |
| S3F2 | NonGM | StageIII | 91 | Panicle development | S5K4 | CK | StageV | 145 | Post-harvest |
| S3F3 | NonGM | StageIII | 91 | Panicle development | S5K5 | CK | StageV | 145 | Post-harvest |
| S3F4 | NonGM | StageIII | 91 | Panicle development | S5T1 | GM | StageV | 145 | Post-harvest |
| S3F5 | NonGM | StageIII | 91 | Panicle development | S5T2 | GM | StageV | 145 | Post-harvest |
| S3K1 | CK | StageIII | 91 | Panicle development | S5T3 | GM | StageV | 145 | Post-harvest |
| S3K2 | CK | StageIII | 91 | Panicle development | S5T4 | GM | StageV | 145 | Post-harvest |
| S3K3 | CK | StageIII | 91 | Panicle development | S5T5 | GM | StageV | 145 | Post-harvest |

Note:^*^ Samples of S1F5, S1K5 and S4K5 were unavailable; ^#^, NonGM, Minghui63 non-transgenic rice; GM, Huahui No. 1 cry1Ab/cry1Ac transgenic rice; CK, blank control.
