# Supplementary figures and images for: Bt-Modified Transgenic Rice May Shift the Composition and Diversity of Rhizosphere Microbiota
Source: Plants (Basel). 2024 May 8;13(10):1300. doi: 10.3390/plants13101300 (PMC11125220; doi:10.3390/plants13101300)

**Repetition: [1.5% of variance; P < NULL; 95% CI = 1%, 2%]**

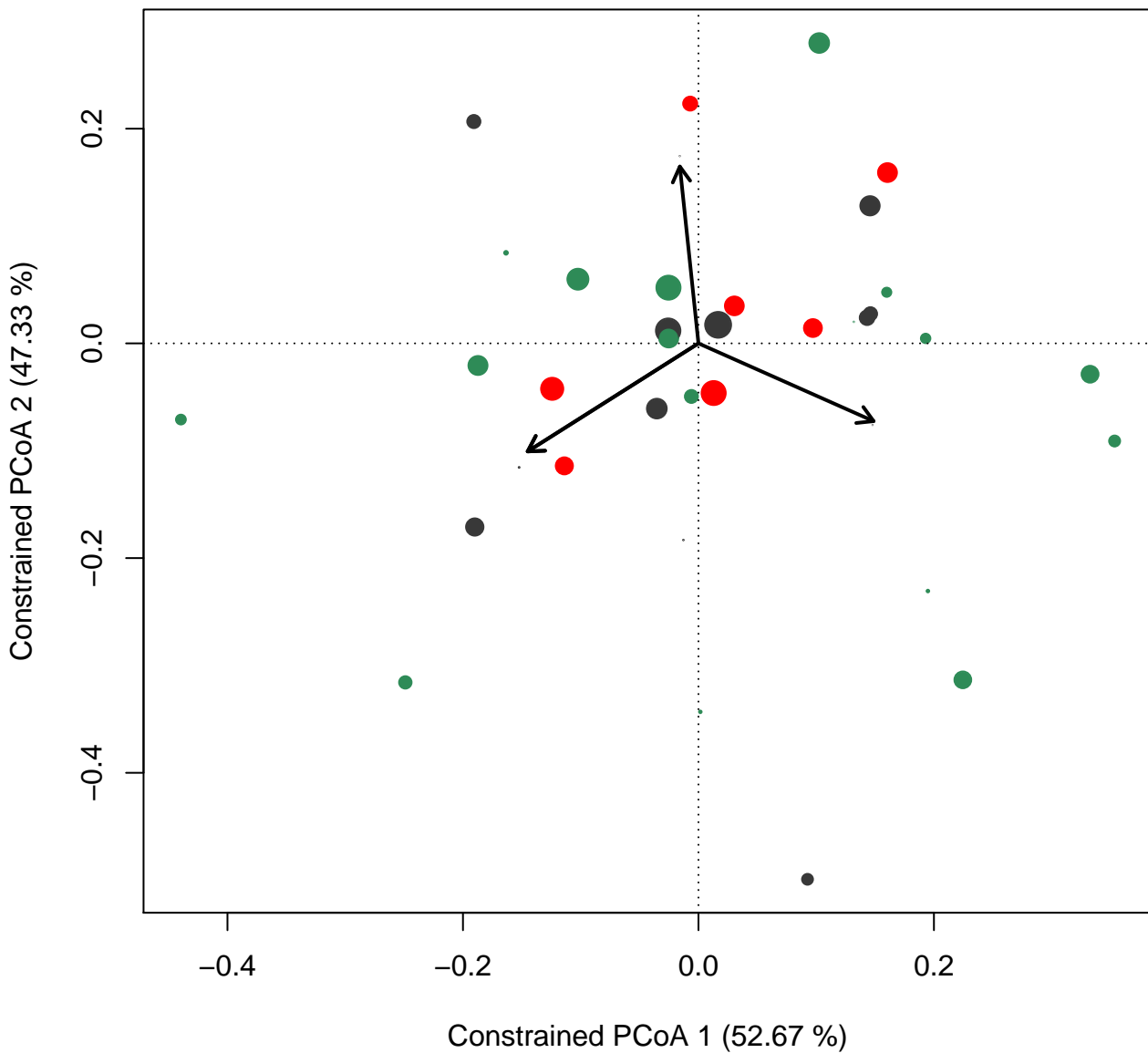

Supplement: Supplementary file 1 [file plants-13-01300-s001.zip › R-scripts/my_outputfiles/Fig_5B.pdf]

Species: [1.5% of variance;  $P < \text{NULL}$ ; 95% CI = 1%, 2%]

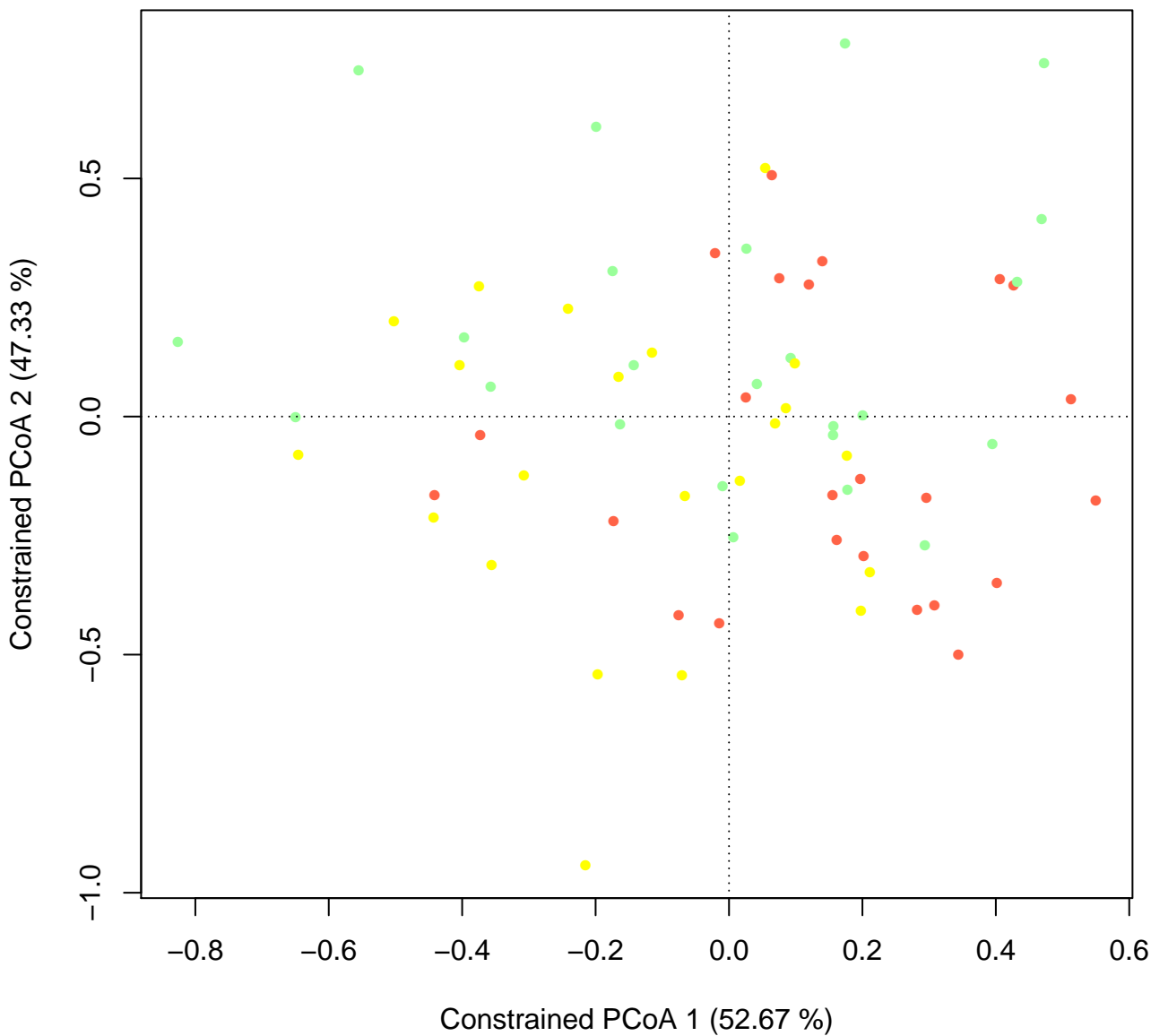

Supplement: Supplementary file 1 [file plants-13-01300-s001.zip › R-scripts/my_outputfiles/Fig_S22.bak.pdf]

Species: [1.5% of variance;  $P < \text{NULL}$ ; 95% CI = 1%, 2%]

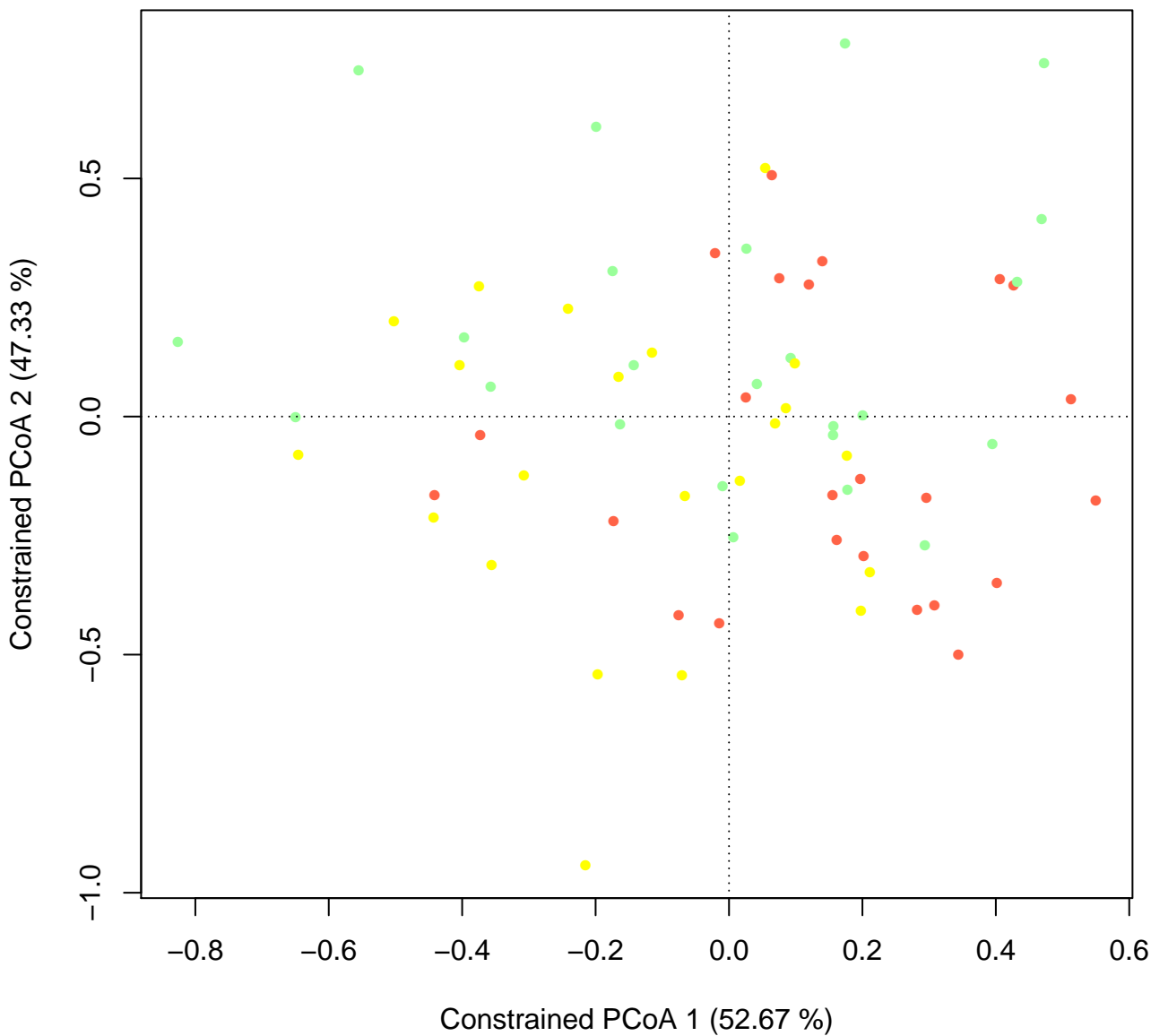

Supplement: Supplementary file 1 [file plants-13-01300-s001.zip › R-scripts/my_outputfiles/Fig_S22.pdf]
